# Concomitant downregulation of neuropeptide genes in a marine snail with consecutive sexual maturation after a nuclear disaster in Japan

**DOI:** 10.3389/fendo.2023.1129666

**Published:** 2023-03-10

**Authors:** Fumihiro Morishita, Toshihiro Horiguchi, Hiroto Akuta, Tatsuya Ueki, Takuya Imamura

**Affiliations:** ^1^ Program of Basic Biology, Graduate School of Integrated Sciences for Life, Hiroshima University, Higashi-Hiroshima, Hiroshima, Japan; ^2^ Department of Biological Science, Faculty of Science, Hiroshima University, Higashi-Hiroshima, Hiroshima, Japan; ^3^ Health and Environmental Risk Division, National Institute for Environmental Studies, Tsukuba, Ibaraki, Japan; ^4^ Program of Biomedical Science, Graduate School of Integrated Sciences for Life, Hiroshima University, Higashi-Hiroshima, Hiroshima, Japan

**Keywords:** transcriptome, reproduction, epigenome, gastropod, precursor, mass spectrometry

## Abstract

Consecutive sexual maturation (CSM), an abnormal reproductive phenomenon of a marine snail, *Reishia clavigera*, has occurred since 2017 in the vicinity of the Fukushima Daiichi Nuclear Power Plant after the nuclear disaster there. We hypothesized that alterations in animal physiology mediated through genetic/epigenetic changes could sensitively reflect environmental pollution. Understanding the mechanism of this rapid biological response should enable us to quantitatively evaluate long-lasting effects of the nuclear disaster. To determine the molecular basis for CSM, we conducted transcriptome profiling in the ganglia of normal and CSM snails. We assembled the short-read cDNA sequences obtained by Illumina sequencing, and succeeded in characterizing more than 60,000 gene models that include 88 kinds of neuropeptide precursors by BLAST search and experimental curation. GO-enrichment analysis of the differentially expressed genes demonstrated that severe downregulation of neuropeptide-related genes occurred concomitantly with CSM. In particular, significant decreases of the transcripts of 37 genes among 88 neuropeptide precursor genes, including those for myomodulin, PentaFVamide, maturation-associated peptide-5A and conopressin, were commonly observed in female and male CSM snails. By contrast, microseminoprotein precursor was the only exceptional case where the expression was increased in CSM snails. These results indicate that down-regulation of neuropeptide precursors is a remarkable feature of CSM. We also found that factors involved in epigenetic modification rather than transcription factors showed altered patterns of expression upon CSM. Comprehensive expression panels of snail neuropeptide precursors made in this study will be useful tools for environmental assessment as well as for studying marine reproductive biology.

## 1 Introduction

Seasonality of reproduction is a key for the conservation of many species. Regulatory systems for reproduction have been evolved to assure sequential occurrence of sexual maturation and behaviors with appropriate timing. These systems are triggered by certain environmental cues, such as temperature, tidal cycle, and day length (1). Historically, reproduction of wild animals has often been threatened by environmental risks imposed by man-made toxic chemicals. For example, in the 70s-80s, populations of mollusks such as bivalves and gastropods declined rapidly, and this decline had an impact on the ecosystem of the intertidal zone and on the fish cultivation industry (2, 3). In gastropods exposed to antifouling bottom paints, secondary formation of male sexual organs in females (imposex) was frequently observed (4) and disturbed normal oviposition. In Japan, using a caenogastropod mollusk such as *Reishia clavigera*, it was elucidated that organotin compounds released from the anti-fouling paint on ships and fishery nets overactivated a nuclear receptor, retinoid X receptor (5), which resulted in the imposex in females (6). Since then, accumulating evidence has prompted worldwide cessation of the use of organotin compounds in anti-fouling paint to try to remediate ecosystems in the intertidal zone. Accordingly, caenogastropods have been focused on as important biomarkers for monitoring the recovery of ecosystems in the intertidal zone from pollution by organotin compounds (7).

The 2011 Great East Japan Earthquake and Tsunami caused multiple environmental risks for the intertidal zone ecosystem on the Pacific coast of eastern Japan. The earthquake and accompanying tsunami totally destroyed more than 100,000 houses, and washed more than 560 km^2^ of maritime area (8). A considerable volume of debris was withdrawn into the sea, which markedly changed the landscape of the intertidal zone, and had negative effects on the ecosystem. More seriously, the earthquake and tsunami destroyed facilities in the Fukushima Daiichi Nuclear Power Plant (FDNPP) and induced melting-down of nuclear reactors. Consequently, as much as several petabecquerels of radioactive substances were released into the environment (9, 10). Most of the radioactive substances have flowed into the ocean through rain, rivers, streams, and underground water, and from the contaminated plume (11).

Field research monitoring the recovery of animal fauna began in late 2011 by establishing several research points on the Pacific coast of Miyagi, Fukushima and Ibaraki prefectures and has continued to date (10, 12). In 2011, impacts on the ecosystem by the nuclear disaster were evident. Diversity of the animal fauna was much lower than that before the disaster; for example, only a few mussels and barnacles were found in the vicinity of FDNPP (within 10 km) (13). Continuous field monitoring revealed that diversity in the ecosystem was restored gradually and returned to the normal level by 2016, in most of the area damaged by the earthquake and tsunami. However, it was still not restored in 2016 around the FDNPP, despite the fact that radioactivity in the environment was below the standard value (10). One possible reason for this delayed recovery may be the continuous cement loading around the harbor of FDNPP, which encloses the sediment containing radioactive materials. Nearly 100,000 tons of cement was thrown into the harbor from March, 2012 to April, 2015 (10). It was estimated that the cement contains tons of chrome, cadmium, arsenic and lead, and a part of it was released into the sea. Therefore, the nuclear disaster seems to have created complex pollution caused by the spread of radioactive substances and heavy metals in the ocean.

The aforementioned marine snail, *R. clavigera*, a common species along the Japanese coast except in Okinawa islands, is an important biomarker for evaluation of environmental pollution in the seashore (14). It became rare to find *Reishia clavigera* from late 2011 to early 2013, probably because most died due to exposure to radioactive substances and subsequent inhibition of larval recruitment by exposure to harmful substances, such as radionuclides and heavy metals. Horiguchi et al. found that, within the area 3-km from FDNPP, the testis and ovary of *R. clavigera* are sexually matured year-round, whereas this marine snail, collected at a reference site, approximately 120 km south of the FDNPP (Hiraiso), normally exhibits gonadal maturation in early summer and regression in winter (15). This unusual phenomenon was named “consecutive sexual maturation (CSM)” (15). Because of the high tidal level and rough waves in winter in that area, it is hard to determine whether the CSM snail lays eggs in mid-winter in that area. However, the latest egg-deposition of *R. clavigera* was observed in late September, which is 2 months later than the spawning season of the normal snail (15).

Recent transcriptome analysis has demonstrated that neuropeptide precursors are up- and down-regulated in association with the mollusk reproduction cycle (16, 17). Several lines of evidence demonstrated that neuropeptides and/or peptide hormones play crucial roles in regulating reproduction in mollusks (18–22). Gonadotropin releasing hormone (GnRH), gonadotropin inhibitory hormone (GnIH) and kisspeptin are key peptides for reproduction in mammals (23) and birds (24), while in mollusks, a peptide hormone, egg-laying hormone (ELH) in *Aplysia*, and myomodulin in Sydney rock oyster (25), induces egg spawning, and GnRH-related peptide induces proliferation of gonadal cells in oyster (26).

Our working hypothesis is that malfunctioning of a regulatory neuropeptide system is associated with CSM. In *R. clavigera*, Leung and colleagues conducted RNA-seq analysis on the animal collected in Hong Kong, China, and identified several neuropeptide precursors (27). In order to test our hypothesis, we decided to further deepen the information on the neuropeptide precursor genes. In this study, we therefore aimed to 1) provide a dataset of genes and peptides expressed in the central nervous system (CNS) ganglia of *Reishia* collected on the coast of Japan through transcriptome analysis, and 2) determine the differences in expression between the normal and CSM snails. Integrated analyses using RNA-seq and Nanoscale-LC-Orbitrap MS/MS provide a comprehensive catalog of mature neuropeptides in *Reishia* ganglia as important biomarkers for the assessment of disaster effects. Here, we provide evidence that a wide range of neuropeptide precursors are downregulated in the CSM snails, indicating the high diversity of this population since the 2011 Great East Japan Earthquake.

## 2 Materials and methods

### 2.1 Animals

The rock shell, *R. clavigera*, were collected on the seashore around Ottozawa, Okuma town (Fukushima, Japan), and Hiraiso, Hitachinaka city (Ibaraki, Japan), respectively, in February, 2021. In February, more than 80 % of *R. clavigera* collected at Okuma were sexually mature (Stage III or IV), whereas none of the animals collected at Hiraiso were sexually mature (they were Stage I or II) (15). Therefore, we refer to *R. clavigera* collected in Hiraiso as the normal snail, and the animals collected in Okuma as the CSM snail, hereafter. Because *R. clavigera* is neither a target species for commercial fisheries, nor protected by the act on conservation of endangered species of wild fauna and flora in Japan, permission is not required for the collection of this marine snail in the field. However, since Ottozawa is located within the restricted area (difficult-to-return zone) in the vicinity of FDNPP, we obtained a permission for entry from the Fukushima Prefectural Government (#2-KIKAN4067). The numbers of animals collected were twenty-nine of CSM snails at Ottozawa, and thirty-five of normal snails at Hiraiso, respectively, which had little influence on the stability of population of *R. clavigera* in those area. Less than ten snails were kept in a 10-litter tank filled with artificial seawater (ASW, Marine Art Hi, Osakayakken Co. Ltd., Osaka, Japan) at 15 °C for a few days until the dissection of ganglia. All the tanks were continuously aerated and filtered the debris with circulation pump. Although the research on the invertebrate does not require animal-ethics approval by the animal ethics committee of Hiroshima University, snails were handled with care to avoid unnecessary stress in accordance with guideline for the animal welfare by the committee.

### 2.2 Transcriptome analysis

#### 2.2.1 Purification of total RNA

The CNS of *R. clavigera* consisted of the circum-esophageal ganglia (the cerebral, buccal, parietal, supra-esophageal, sub-esophageal and pedal ganglia) in the head region and the visceral ganglion located near the heart. Both the circum-esophageal and visceral ganglia were dissected from animals collected at Hiraiso (normal snails) and Okuma (CSM snails) for the purification of total RNA for RNA-seq. The marine snails were placed in 0.33 M MgCl_2_ for muscular relaxation before the dissection. Each dissected CNS was immediately frozen in liquid nitrogen (LN_2_). Frozen tissues from each snail were placed in a separate plastic 1.5 ml tubes and preserved in a deep freezer at -75 °C.

Of the ganglia dissected, 9 of CMS snails (male 4, female 5) and 9 of normal snails (male 5, female 4) were used for the RNA extraction for RNA-seq analysis. Rest of frozen ganglia were preserved for pilot experiments. Total RNA was extracted from each ganglion separately. For the extraction, each ganglion was placed inside of a folded aluminum foil and put on an aluminum block, and then crushed with a hammer. All of the devices had been chilled in LN_2_. Powdered frozen tissue was thawed in 0.2 ml of Sepazol RNA I Super G (Nacarai Tesque Inc., Kyoto, Japan), and homogenized by gentle pumping with a micro-pipet. Total RNA was extracted from the lysate according to the manufacturer’s instruction. DNA remaining in the total RNA sample was removed by DNase digestion as follows: the pellet was dissolved in 0.2 ml of buffer RA1 supplied with a column kit, NucleoSpin RNA XS (Macherey-Nagel GmbH & Co. KG, Duren, Germany), and then mixed with an equal volume of 70 % ethanol by pipetting. Then, it was loaded on the NucleoSpin RNA XS column, and DNase digestion was conducted according to the manufacturer’s instructions. Total RNA was eluted from the column with 15 µl of RNase-free water, and was quantified with a NanoDrop 1000 spectrophotometer (Thermo Fisher Scientific K.K., Tokyo, Japan). Qualitative analysis of purified RNA was conducted with a Bioanalyzer (Agilent Technologies Ltd., Santa Clara, CA) by the Natural Science Center for Basic Research and Development, Hiroshima University.

#### 2.2.2 Construction of RNA-library for Illumina

Of the RNA samples extracted, total seven samples of female snails (four of normal and three of CSM) with high yield and RNA integrity number were used for the RNA-library construction. An RNA-library for Illumina sequencing was constructed with the NEBNext Ultra Directional RNA Library Prep Kit for Illumina or NEBNext Ultra II Directional RNA Library Prep Kit for Illumina (New England Biolabs Japan, Tokyo, Japan), according to the manufacturer’s manual, using 200-250 ng of total RNA for the starting materials. These libraries were labeled with different indexes. The library obtained was analyzed using the Bioanalyzer to confirm that there was no bias in the distribution of fragment cDNA size. The average size of the cDNA was 351-411 bp. The concentration of cDNA in the library was determined by following the qPCR-based protocol with the GenNext NGS library quantification kit for Illumina (TOYOBO Co. Ltd., Osaka, Japan). They were adjusted to 5 nM and all of them were mixed together (total 30 μl) for a single analysis. For the RNA-seq analysis on male snails, six libraries (three each from normal and CSM snails) were constructed, similarly. The average size of cDNA was 372-412 bp. The obtained libraries were mixed using different volumes that were chosen so that the difference in the final concentrations was minimized. The male and female libraries were sent to Macrogen Japan Corp. (Tokyo, Japan), and sequenced with a HighSeq X (150 bp paired end), separately.

#### 2.2.3 Data analysis for RNA-seq

**Figure 1:**
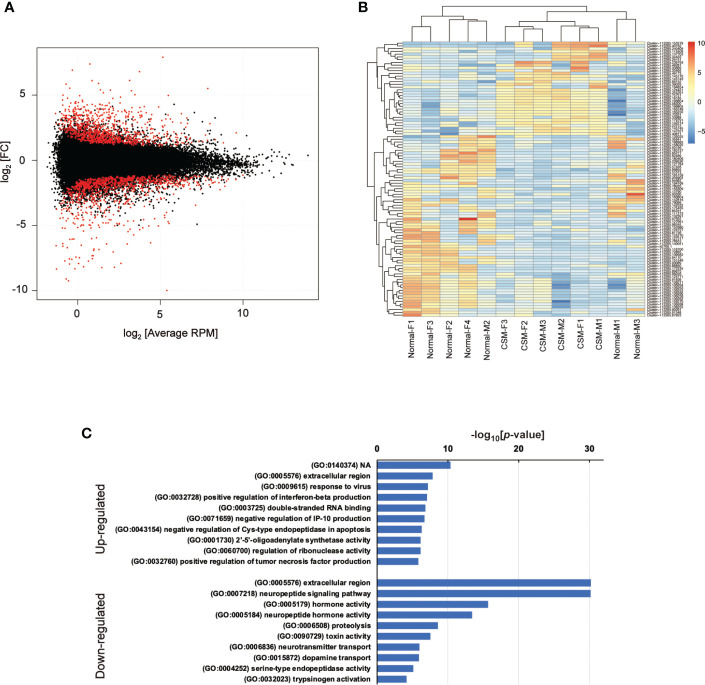
Summary of RNA-Seq analysis on *Reishia* ganglia. **(A)** MA-plot for the merged clusters obtained by RNA-seq analysis of male and female *Reishia* CNS. Differentially expressed clusters between the normal and CSM snails with FDR below 0.05 are indicated by red dots. RPM: reads per million mapped reads, FC: fold change. **(B)** Heatmap of top 100 DEGs between the CNS of normal and CSM snails. DEGs between the normal and CSM snails were extracted for males and females by calculating the log_2_ value relative to average RPM, and the top 100 DEGs were represented by color. **(C)** GO-enrichment analysis of DEGs in *Reishia* CNS. GO-enrichment analysis was conducted on 253 clusters of up-regulated genes and 334 clusters of down-regulated genes, and the top 10 of enriched GO-terms for up-regulated (upper) and those for down-regulated (lower) are indicated. Note that, in down-regulated genes, GO-terms associated with neuropeptide signaling are enriched.

Because genome sequence data for *R. clavigera* is not available, short read sequences obtained by NGS sequencing were assembled into clusters as follows. First of all, adaptor sequences were trimmed by Trim Garore (ver 0.6.6), and then, the short-read sequences were assembled by Trinity (ver. 1.12.0). The assembled sequences were mapped on the model transcript made with Salmon (ver 1.5.2). The model transcripts were refined by removing duplicates and re-mapping on the original model transcript using seqkit (ver 0.15.0). Model transcripts were further clustered by corset (ver 1.09), and annotated using BLAST search on GenBank. To determine the changes in gene expression by CSM, differences in expression levels of the respective gene models between normal and CSM snails were calculated using edgeR (ver 3.34.0), and 253 of up-regulated and 334 of down-regulated models were extracted as the differentially expressed genes (DEGs) with false discovery rate (FDR) less than 0.05 (**Figure 1A**).

### 2.3 Identification of neuropeptide precursors

For the identification of neuropeptide precursors, a command-line tool for running BLAST, BLAST+, was installed. Then, the translated clusters were searched by TBLASTN (2.12.0) using the amino-acid sequences of neuropeptide precursors of a giant triton snail, *Charonia tritonis* (28), as well as several precursors of insects, as queries. Precursor proteins encoded by the retrieved clusters were analyzed with SignalP (ver. 4.1) for prediction of N-terminal signal peptides, and with NeuroPred for prediction of processing sites.

### 2.4 Identification of neuropeptides by mass-spectrometry

Peptides in CNS of the normal snails were identified by mass spectrometry-based *de novo* sequencing of peptides. Crude peptidic extract was prepared from 25 pooled ganglia of male and female of normal *R. clavigera*. Dissected CNS was frozen quickly in LN_2_, and preserved at -75 °C. On the day of extraction, frozen tissues were powdered in LN_2_, then transferred slowly into boiling pure water (3 ml), and kept boiling for more than 5 min. The extract was acidified by adding acetic acid at final concentration 5 %, and then homogenized with a Teflon-glass homogenizer. The homogenate was centrifuged at 20,000 x g for 10 min, and the supernatant was collected. The pellet was resuspended in 5 % acetic acid, and then subjected to homogenization and centrifugation as described above. The two extracts were combined, and concentrated to 100 µl.

For the de-salting, the concentrated extract was loaded on a C_18_ Sep-Pak Plus column (Waters Corporation, Milford, MA), that had been equilibrated with 0.1 % trifluoroacetic acid (TFA). After the washing with 0.1 % TFA, retained materials were eluted stepwise with 20 %, 40 % and 60 % acetonitrile with 0.1 % TFA. Eluted materials were combined and condensed to 0.1 ml with a vacuum centrifuge. This crude extract of *Reishia* CNS was filtered through a 0.45 µm filter, and then subjected to shot-gun analysis with a nanoLC-Orbitrap mass spectrometer.

Mass spectrometry was conducted by the Natural Science Center for Basic Research and Development, Hiroshima University. One microliter of the extract was mixed with 14 μl of 0.1 % formic acid. One microliter of the diluted sample was injected into the nano-scale liquid chromatography system (Ultimate 3000 RSLCnano, Thermo Fisher Scientific, Germering, Germany) equipped with a trap column (Accliam PepMap100 C_18_, I.D 300 μm x 5 mm, Thermo Fisher Scientific K.K., Tokyo, Japan) and a separation column (Nikkyo nano HPLC capillary column, 3 µm C_18,_ I.D. 75 µm x 120 mm, Nikkyo Technos Co. Ltd., Tokyo, Japan), connected in tandem. Retained materials were eluted using a linear gradient of acetonitrile (4-35 % over 100 min, 200 nl/min), and then directly ionized by nano-scale electrospray at source voltage between 1.5 and 2.5 kV.

The mass spectrum for the precursor ion was obtained using an LTQ-Orbitrap XL mass spectrometer (Thermo Fisher Scientific K.K., Bremen, Germany) with Fourier transform mass spectrometry mode. Scanning was conducted with scan range from 200-1,500 (m/z) and resolution at 30,000. Then, the parent ion was fragmented by collision with helium gas with collision energy of 35 and isolation width of 2.0 (m/z). The conditions for mass measurement of fragment ions were the same as that for the parent ion. Obtained mass data were analyzed using an application, PEAKS X (Bioinformatics Solutions Inc., Waterloo, Canada), for *de novo* sequencing. For the further identification of translated products of neuropeptide-precursor genes, BLAST search using the fragment peptides identified by mass-spectrometry as query was conducted on the dataset of amino-acid sequences of neuropeptide precursors obtained by RNA-seq.

## 3 Results

### 3.1 *De novo* assembly of RNA-seq data of the central ganglia of *Reishia clavigera*

CSM is more evident in females than in males (15). First, seven cDNA libraries prepared from four normal female and three CSM female central ganglia were subjected to a single-run of Illumina-sequencing, and a total of 384,117,268 reads of short-read DNA sequences were obtained. Incomplete sequences and index sequences on each sequence were removed by Trim Garore, which recovered 97.5 % of the original short-read DNA sequences. Because genome sequence data for *R. clavigera* is not available, a reference transcript model was made by assembling those short-read DNA sequences by Trinity and corset. We finally obtained 67,481 annotated reference gene models. An MA-plot to compare CSM and normal females indicated that read normalization was successfully done for the identification of differentially expressed genes (DEGs: **Figure S1**). A heatmap of the top 100 DEGs demonstrated that about 1/3 of them were up-regulated, while the remaining ones were down-regulated, in CSM snails (**Figure S2**). When the short-read DNA sequences were mapped on these gene models, 88.7 % of the short-read DNA sequences were successfully mapped. We evaluated the accuracy and redundancy of the reference models using BUSCO, and found that 84.2 % of the model was matched to the core-gene set of mollusk (Mollusca_odb10). Thus, we concluded that our protocol for the cluster assembling for a reference transcript model worked well with the RNA-seq data obtained from female *Reishia* ganglia.

Similar analyses were applied for cDNA libraries of male central ganglia (three from normal, and three from CSM), and short read sequences obtained (total 384,117,268) were processed. The hierarchical clustering of gene expressions of male and female central ganglia demonstrated that the thirteen samples were clustered based on maturation status, not sex (**Figure S3**). Thus, occurrence of CSM after the East Japan Great Earthquake has had considerable impacts beyond sex difference, suggesting that sex-independent mechanisms might mediate some of the transcriptome alterations. Then, we combined the short-read DNA sequences obtained from females and males together, performed re-assembling of short-read DNA sequences, and finally identified 350,645 reference transcript models from RNA-seq data of *Reishia* ganglia. Consequently, comparison of the gene model with a 5,295 core-gene set of mollusk (Mollusca_odb10) by BUSCO ended with 91.5 % match, including 68.6 % of single copy genes and 15.6 % of duplicated genes, supporting the high quality of our gene models.

### 3.2 Identification of differentially expressed genes between the normal and CSM ganglia

Using the refined transcript models, the short-read DNA sequences obtained from females and males were mapped again, and differentially expressed genes between the normal and CSM ganglia were extracted (**Figure 1**). The heat map constructed with the top 100 highly up- or down-regulated genes is shown in **Figure 1B**. GO-term enrichment analysis revealed that up-regulated transcript models were characterized by GO-term such as extracellular region (GO: 0005576) and response to virus (GO:0009615), but not by neuropeptide related precursors (**Figure 1C**). Up-regulated genes that include 26 cell signaling associated genes are summarized in **Table S1**. Cell signaling associated genes were further classified into 12 of signal transduction-associated genes such as small GTPase, 9 of receptors such as Tyr-kinase and GPCR and two genes of ligands. The BLAST search demonstrated that this ligand has similarity to a precursor of beta-microseminoprotein (MSP). MSP was originally identified as a small proteinaceous pheromone, synthesized and released from the mammalian prostate gland into seminal fluid. A structurally related peptide has been found in the egg-mass of a cephalopod. In this context, MSP is a reproduction-associated peptide. To our knowledge, this is the first identification of MSP in a molluscan brain.

In contrast to the data on upregulated genes, GO-enrichment analysis on down-regulated genes revealed that GO-terms related to peptide hormones, such as neuropeptide signaling pathway (GO: 0007218), hormone activity (GO: 0005176) and peptide hormone activity (GO:0005184) are enriched, raising the possibility that expression of neuropeptide and/or peptide hormone precursors are totally down-regulated in CSM snails (**Figure 1C**). In fact, down-regulated genes included those showing high similarity with precursors for several molluscan neuropeptides such as FMRFamide, myomodulin and feeding-circuit activating peptide (FCAP). Other down-regulated genes are summarized in **Table S2**.

### 3.3 Identification of neuropeptide precursor in *R. clavigera*

Based on the GO data, we decided to examine the possible concomitant down-regulation of neuropeptides. Therefore, we aimed to construct a more comprehensive catalog of mature neuropeptides in *Reishia* ganglia as important biomarkers for the further assessment of disaster effects. For better identification of neuropeptide precursors from the reference transcript models of *R. clavigera*, we manually searched the translated sequences of the reference models using amino acid sequences of neuropeptide precursors identified in the transcriptome analysis of the giant triton snail, *Charonia tritonis* (28) as queries, and finally increased the coverage up to 88 neuropeptide-encoding genes.

These 88 genes include all the neuropeptide precursors highly homologous to those in *C. tritonis*, except for those of Bursicon-α and -β, from the transcript models of *R. clavigera* with *p*-values mostly less than 1x E^-10^ (**Table S3**). Several genes encode neuropeptide precursors showing high similarity with those found in other gastropods, *Aplysia* and *Lymnaea*, such as *Achatina* cardioexcitatory peptide (ACEP), Clionin and Sensorin. In an abalone, *Haliotis discus hannai*, Kim et al. identified eight novel neuropeptide precursors, maturation associated peptide (MAP) 1-8, which were up- and down-regulated in association with sexual maturation (17). Our analysis identified six MAPs in *R. clavigera*, among which MAP-4 and -7 were not included. Moreover, BLAST search using some neuropeptide precursors of insects and crustaceans identified the precursors for leucokinin and neuroparsin. In cases in which the homology search using a single peptide precursor as the query retrieved two similar but different precursors from the transcript models, such precursors were independently named using numbering or letters of the alphabet, resulting in names such as Elevenin-1 and Elevenin-2, or MAP-1A and MAP-1B. We identified neuropeptide-F precursor as well, which had not been identified in another caenogastropod, *Charonia tritonis*. Moreover, as mentioned above, we identified a precursor encoding microseminoprotein (MSP)-like peptide by BLAST search on GenBank. Identified precursors included several reproduction-associated peptides, such as FMRFamide/FLRFamide, PentaFVamide, Egg-laying hormone (ELH), APGWamide-1/-2, Achatin-1 and Myomodulin (**Figure S4**).

### 3.4 Identification of mature peptides encoded on the precursor

We next examined if the peptides on the respective precursors are processed to become mature peptides through post-translational modification. By predicting processing sites on the precursor using NeuroPred, and considering the fact that the Gly residue just on the N-terminal side of the predicted processing site offers a C-terminal amide, we predicted 327 mature peptides with or without C-terminal amidation from 88 of the precursors (**Figure S4**). Then, we attempted to identify these predicted peptides in *Reishia* ganglia by mass spectrometry. For the *de novo* identification of the peptides, Peaks X was used to predict several modifications of peptides, such as pyro-glutamination on N-terminal Gln or Glu residue, disulfide bond between a pair of Cys-residues, as well as the oxidation of Met residues.

Our peptide sequencing identified 147 predicted mature peptides (45.0%) from 44 precursors (**Table S4**). Several precursors, such as ACEP, Adipokinetic hormone (AKH), Cholecystokinin (CCK)-2, Clionin-1/-2, Elevenin-1/-2, MAP-8, Tetradecapeptide (TDP)-1/-2, *Thais* excitatory peptide (TEP)-1/-2 (note that names of some previously identified peptides include *Thais*, the former name of the genus *Reishia*, and have been still conventionally used as formal ones) and Whitnin, have a single predicted mature peptide (**Figure S4**). Our sequencing identified mature peptides encoded by the precursors of ACEP, AKH, MAP-8, TDP-1/-2 and Whitnin (**Table S4**). Other precursors were predicted to contain multiple copies of the same or structurally related peptides in tandem (**Figure S4**). We identified all or most of the predicted mature peptides encoded by the precursors of Allatostatin-A1/-A2, Buccalin, FLRFamide/FMRFamide, FMRG, FRFamide, FRYamide, FVRIa-1/-2, FXXFamide, GDPFLRFamide, Helicostatin-related peptide (RP), *Helix* command-specific (HCS) 2, Myomodulin, Pedal peptide, PentaFVamide, Small cardioactive peptide (SCP)-1, Tachykinin-1/-2, *Thais* Enterin related peptide (TERP)-1/-2 and WWamide (**Table S4**). However, only one or a few mature peptides were identified in the precursors of SGLVamide, Cerebrin, Crustacean cardioactive peptide (CCAP), GnRH-RP, leucokinin, MAP-5A, MAP-6B, *Mytilus* inhibitory peptide (MIP)-1, Orcokinin-RP, Pleurin, Sensorin-A1/-A2, and VHGY (**Table S4**). Moreover, fragment peptides derived from the precursors of APGWa-2, ELH, Fulicin, GGKVamide, Prohormone-4B and SPamide were also identified by *de novo* sequencing (**Table S5**). Therefore, these precursors were translated and subjected to the post-translational modification in *Reishia* ganglia.

### 3.5 Expression levels of neuropeptide precursors in normal ganglia

**Figure 2:**
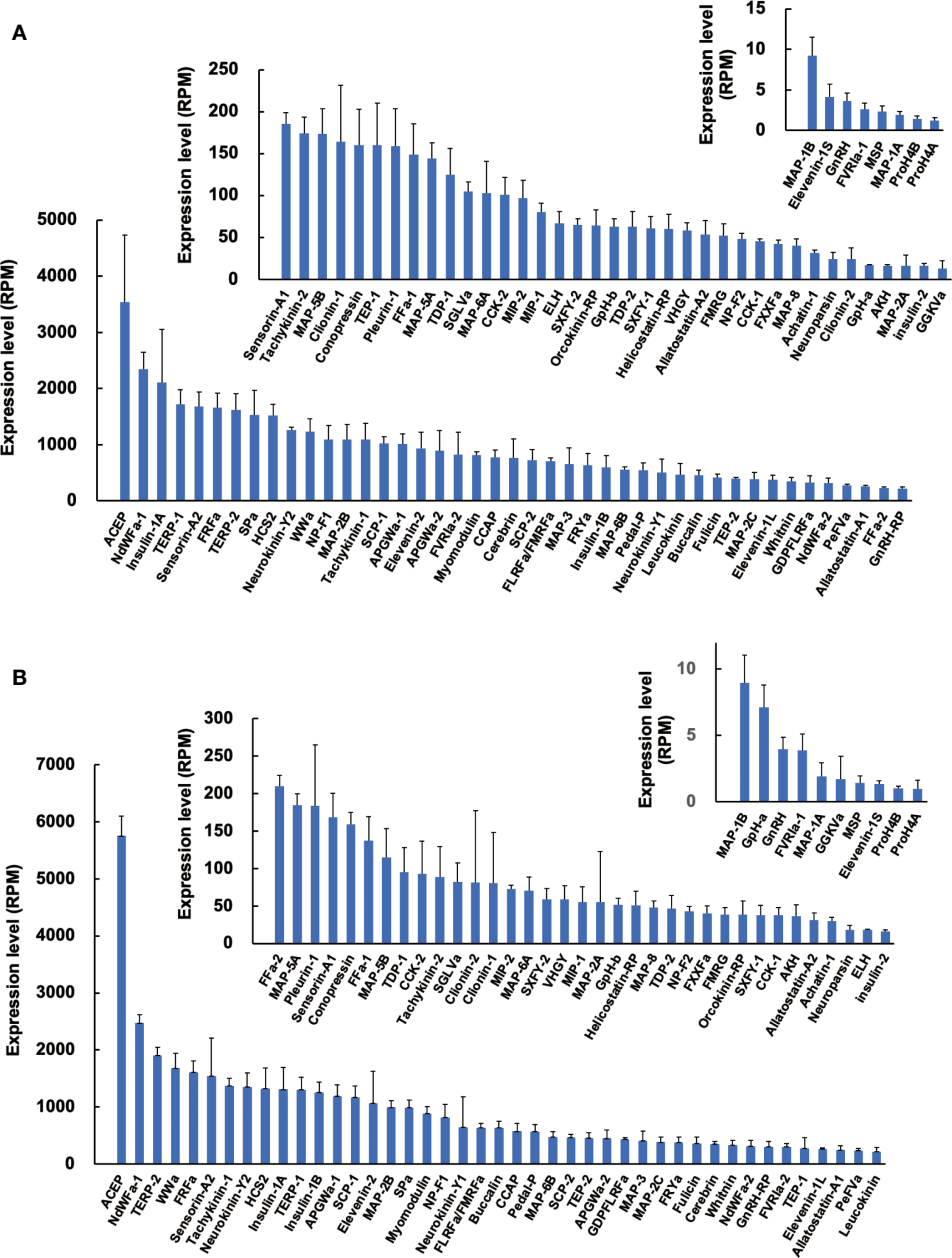
Expression levels of neuropeptide precursors in CNS of normal snail. Expression levels of neuropeptide precursors in female **(A)** and male **(B)** were expressed in RPM determined by RNA-seq analysis. Each column and vertical bar represent mean ± S.E., respectively (N = 4 for female, N = 3 for male). For the abbreviated names of neuropeptides, refer to **Table S3**.

Since precursors for neuropeptides were identified in *Reishia* CNS, we next examined the expression levels of identified precursors by comparison with RPM (reads per million-reads) of respective precursors in normal snails. The most abundantly expressed neuropeptide was ACEP (29) in both the normal male and normal female (**Figure 2**). Average RPM was ca. 3,500 for female (n = 4) and ca. 5,800 for male (n = 3), respectively. ACEP is a potent cardio-excitatory neuropeptide that was originally identified in a giant African snail, *Achatina fulica* (30). ACEP identified from mollusks share the identical WRPQGRF-NH_2_ in C-terminus. Although ACEP precursors have been identified from several animal phyla (31), information on their physiological functions, except for cardiac regulation (30), are largely unknown. By contrast, RPM for several neuropeptide precursors, such as MAP-1A/B, MSP, GnRH, and neuropeptides differ markedly, with up to a few thousand-fold differences.

Comparison of expression levels between males and females demonstrated that expression of MAP-2A is 4-fold higher in males, while that of ELH is 4-fold higher in females. However, sexual differences for other neuropeptide precursors were mostly less than 2-fold. Thus, sexual difference in neuropeptide precursor expression does not seem evident in normal snails.

### 3.6 Difference in expression of neuropeptide precursors between normal and CSM snails

**Figure 3:**
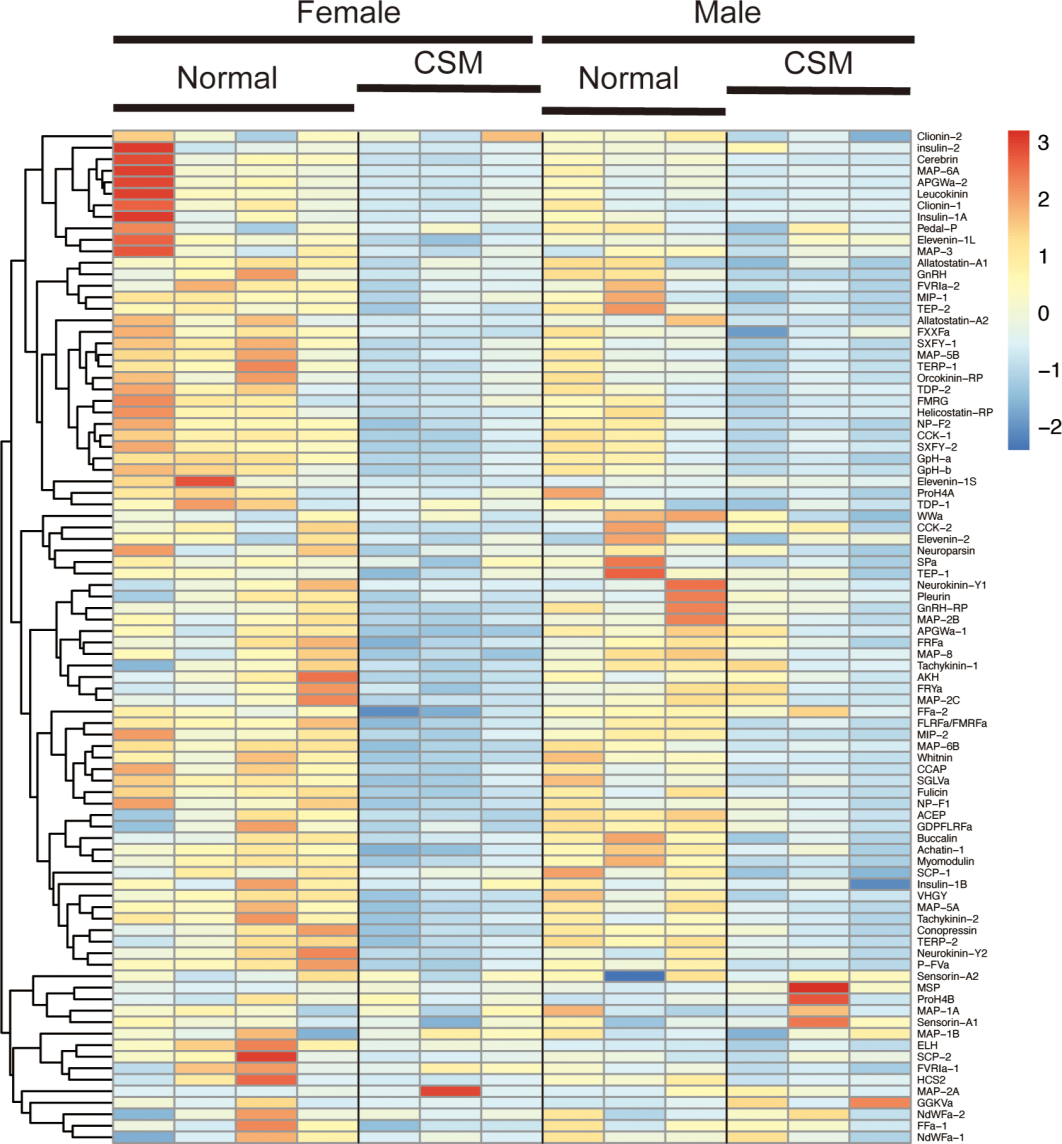
Hierarchically clustered heatmap showing the expression of neuropeptide precursor in the ganglia of the normal and CSM snails. Heatmap representation of differences in expression levels of neuropeptide precursors between the normal and CSM snails was made by calculating the Z-score. Note that over all expression levels of neuropeptide precursors in CSM snails were lower than those in normal snail.

**Figure 4:**
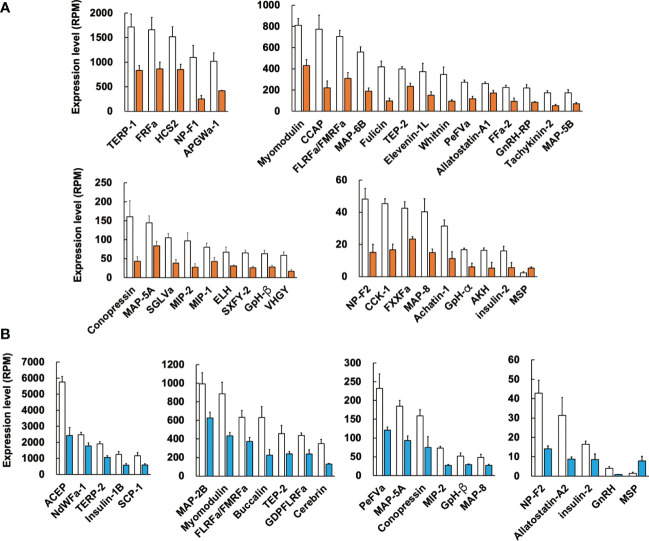
Difference in expression levels of neuropeptide precursors between the normal and CSM snails. RPM of identified neuropeptide precursors in female **(A)** and male **(B)** were compared between the normal and CSM snails, and precursors with statistically significant change (*p* < 0.05 by student’s *t*-test) are indicated. Note that expression of MSP precursor was up-regulated, while other precursors are down-regulated. For the abbreviated names of neuropeptides, refer to **Table S3**. Each column and vertical bar represent mean RPM ± S.E. (n = 3), respectively. Open columns: normal snail, colored columns: CSM snail.

We next examined the difference in expression levels of respective neuropeptide precursors in *Reishia* CNS between the normal and CSM snails. In agreement with the GO-enrichment analysis, the heatmap for the neuropeptide precursors demonstrated that many neuropeptide precursors were down-regulated in the ganglion of the CSM snail (**Figure 3**). **Figure 4** shows averaged RPM for neuropeptide precursors that are significantly down-regulated in CSM snails (*p* < 0.05).

**Figure 5:**
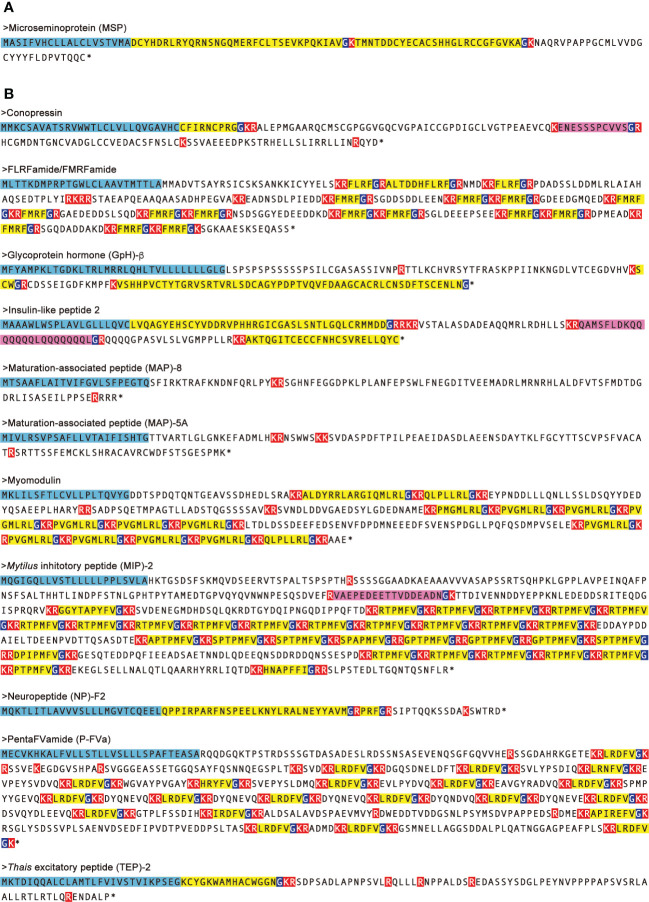
Amino-acid sequences of neuropeptide precursors which are up- and down-regulated in both male and female CSM snails. Neuropeptide precursors up- **(A)** and down- **(B)** regulated in both of male and female CSM snails were indicated. Amino-acid sequences of the precursors were predicted by translating nucleotide sequences of transcript models assembled using RNA-Seq data. N-terminal signal peptides are highlighted with light blue. Predicted processing sites and Gly residues for amide-donner are highlighted with red and dark blue, respectively. Predicted mature peptides are highlighted with yellow. Unique peptides in the precursor are highlighted with magenta. Asterisks represent that amino acid sequence was terminated by stop codon.

In female CSM snails, as many as 37 neuropeptide precursors were significantly down-regulated. The highest fold-change of PRM was found for precursors for Fulicin-1 and Neuropeptide-F2, which were less than 1/4 the levels in normal snails (**Figure 4A**). Other neuropeptide precursors are down-regulated to less than half of the normal snail. In male CSM snails, 22 neuropeptide precursors were significantly down-regulated. The highest fold-change of RPM was found for the precursor of GnRH, whose level was less than 1/4 the level in normal males (**Figure 4B**). Eight other kinds of neuropeptide precursors were down-regulated to less than half the level in normal snails. Thus, down-regulation of neuropeptide precursors was more evident in female than in male snails. Nevertheless, down-regulation of precursors for Conopressin, FLRFamide/FMRFamide, Glycoprotein-β, Insulin-like peptide 2, Myomodulin, MAP-5A, MAP-8, MIP-2, Neuropeptide-F2, PentaFVamide and TEP-2 was seen in both male and female snails. Amino-acid sequences of these neuropeptide precursors are shown in **Figure 5**.

Again, the comparison of RPM between the normal and CSM snails revealed that the expression level of MSP was up-regulated in CSM snails. Although its expression level is quite low in *Reishia* CNS, fold-changes of the expression level of MSP in CSM snails were 2-fold in females, and 4-fold in males.

### 3.7 A possible mechanism by which expression of neuropeptide precursors was modified in the CSM snail

**Figure 6:**
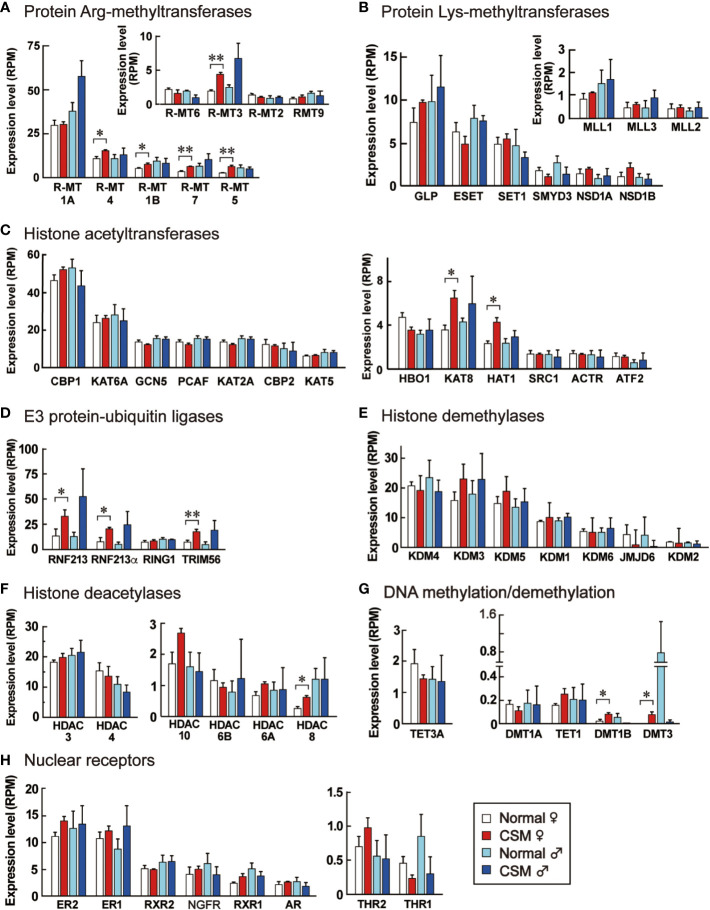
Expression levels of enzymes for epigenetic modification and nuclear receptors in the ganglia of normal and CSM snails. RPM of enzymes for epigenetic modifications and nuclear receptors expressed in Reishia ganglia are shown. They were grouped into protein Arg-methyltransferases **(A)**, protein Lys-methyltransferases **(B)**, histone acetyltransferases **(C)**, E3 protein-ubiquitin ligases **(D)**, histone demethylases **(E)**, histone deacetylases **(F)**, enzymes for DNA methylation and demethylation **(G)**, and nuclear receptors **(H)**, respectively. For the abbreviated names of enzymes, refer to **Table S6**. Each column and vertical bar represent mean RPM + S.E. (n = 3). Open columns: normal female, red columns: CSM female, light blue columns: normal male, blue columns: CSM male. Statistical significance: *: *p* < 0.05, **: *p* < 0.01 by Student’s t-test.

Our RNA-seq analysis demonstrated that down-regulation of neuropeptide precursors is characteristic of CSM snails. To elucidate possible mechanisms that reduced the expression of neuropeptide precursors in CSM, we focused on reconstructing gene models involving nuclear receptors and epigenetic factors. Using a method similar to that we used for identifying the neuropeptide genes, we found the homologous proteins to each query with *p*-value less than 1xE^-20^ (**Table S6**). These proteins included eighteen histone methyltransferases, seven histone demethylases, thirteen histone acetylases, six histone deacetylases, three DNA methyltransferases, two DNA demethylase and eight nuclear receptors (**Table S6**). **Figure 6** shows their expression in normal and CSM snails. RPMs for those proteins are mostly less than 30, suggesting that expression levels of these proteins are relatively low in the central ganglia. Especially, expression levels of enzymes for DNA methylation and demethylation are negligible. Nevertheless, in the female snail, expression levels for five histone arginine-methyltransferases, but not histone lysine-methyltransferases, two histone acetyltransferases, two DNA methyltransferases and three E3 ubiquitin-protein ligases are significantly up-regulated (*p* < 0.05). These enzymes promote chemical modification of histone and DNA. Histone-deacetylase (HDAC) 8, that catalyzes deacetylation of histone, was the only example up-regulated in CSM female snail. No changes in expression levels between normal and CSM snails were found for other histone demethylases, histone deacetylases or nuclear receptors examined.

## 4 Discussion

### 4.1 Consecutive sexual maturation in *Reishia clavigera*

CSM is an abnormal reproductive phenomenon exhibited by a marine snail, *R. clavigera*, found in the vicinity of FDNPP in Japan. In general, animals spend much energy for gonadal maturation (32). CSM would be expected to have a negative effect on the recovery of the population of *R. clavigera*, because this marine snail would consume much energy for maintenance of the mature gonad throughout the year, despite the fact that half of the year is not suitable for survival of hatched embryos. Because CSM has recently been found in this marine snail for more than 2 years, it seems that CSM has been occurring continuously, not temporally. This suggests that the nuclear disaster might have resulted in a novel population of *R. clavigera* that has lost the seasonality of reproduction. Elucidating the molecular mechanism of CSM is an urgent task for understanding the impact of the nuclear disaster in FDNPP on the marine ecosystem, with the ultimate aim of designing of an effective approach for remediation of the marine environment.

### 4.2 Concomitant down-regulation of neuropeptide precursors in CSM snails

In this study, we attempted to overview the comprehensive changes in gene expression in CSM snails by RNA-Seq analysis. Our assembly of short-read cDNA sequences obtained by the Illumina sequencer successfully identified more than 60,000 kinds of gene models in *Reishia* ganglia. Among them, we identified as many as 88 kinds of neuropeptide precursors.

Remarkably, of the 88 neuropeptide precursors in this marine snail, 36 were down-regulated in female, while 22 precursors were down-regulated in male. Such a concomitant down-regulation of neuropeptide precursors influences the precursors of Conopressin, FLRFa/FMRFa, Glycoprotein-β, insulin-like 2, MAP-5A, MAP-8, MIP-2, Myomodulin, Neuropeptide-F2, PentaFVamide and TEP-2 in both males and females. It has been reported that myomodulin, FMRFamide and conopressin are localized in the penis nerve, and modulate the motility of the penial complex in a freshwater snail, *Lymanea stagnalis* (33, 34). Expression levels of MAP-5A and MAP-8 are up-regulated in association with reproduction of a pacific abalone, *Haliotis discus hannai* (17). Although the exact physiological functions of these peptides in *R. clavigera* remain unknown, we assume that these neuropeptides mediate reproduction also in *R. clavigera*, which was affected by the nuclear disaster, leading to CSM in *R. clavigera*.

Such a concomitant down-regulation of neuropeptides also caused a considerable impact on development of a monitoring system that might aid in recovery from the disaster. In this context, it is interesting to note that several precursors, such as ACEP in male snails, and CCAP and Fulicin in females, showed high expression levels in the normal snail, while they showed markedly decreased expression levels in the CSM snail. For example, CCAP in female snails shows expression levels of 800 RPM, and the expression decreases by 25% upon CSM. Therefore, our data enables the establishment of an easy, rapid and sensitive detection system to assess environmental pollution.

### 4.3 How is the down-regulation of neuropeptide precursors involved in the CSM of *R. clavigera*?

Assuming that down-regulated neuropeptides mediate reproduction in this marine snail, how are they involved in CSM? We have previously reported that TEP-2 augments the motility of the penial complex in *R. clavigera* (35). However, to our knowledge, TEP-2 is the only peptide that has been tested to reveal its physiological function in *R. clavigera*. Therefore, the expression patterns of neuropeptides identified in this study provide a key for estimating the roles of the respective neuropeptides. Réalis-Doyelle et al. (16) conducted transcriptome analysis on Pacific oyster at different gonadal maturation stages, and identified four representative patterns of changes in expression of neuropeptide precursors. Interestingly, several peptides, including MIP, prothoracicotropic hormone and RXIamide are up-regulated in the early phase of gonadal maturation, and then down-regulated in the later phase. These peptides are assumed to be involved in the regulation of proliferation of the germ-cell line and/or of the energy metabolism for reproduction (16). By analogy to this fact, it is interesting to assume that CSM snails do not need to make a gonadal system to be mature, and therefore, the related neuropeptides are totally down-regulated, as we clearly observed in the RNA-seq data.

Among the neuropeptides down-regulated upon CSM, some seem to function in the inhibition of gonadal maturation, such as GnIH, which has been found in birds (36), C-type natriuretic peptide, found in mammalian follicle granulosa cells (37), and proteinous reproduction-inhibitory factors found in a freshwater snail, *Lymnaea stagnalis* (38), and a scallop, *Patinopecten yessoensis* (39). Such maturation-inhibitory peptides should increase in seasons that are not suitable for reproduction, such as winter. Testing whether the administration of these peptides can rescue *R. clavigera* from CSM is our next challenge toward elucidating the molecular mechanism of CSM.

### 4.4 How is concomitant down-regulation of neuropeptides achieved in CSM snails?

We do not yet know the mechanism of totally down-regulating neuropeptides in CSM snails. To our knowledge, this is the first report of animals that show concomitant down-regulation of neuropeptides. There should be *trans*-acting factors that bind to *cis*-regulatory regions frequently found in neuropeptide genes (40). It is known that cAMP-responsive elements are prevalent in neuropeptide precursor genes and are thought to function in the up-regulation of these genes in many species. However, we could not find down-regulated factors that can bind to cAMP-responsive elements (**Figure 6**). In the case of pollution driven by organotin compounds, RXR, a retinoid X receptor, has frequently been observed to be up-regulated in several tissues, including the CNS (41). However, we could not find down-regulation of nuclear receptor genes, including RXR (**Figure 6**).

Our transcriptome analysis also provided a clue about the mechanism that modified the gene expression of precursors for neuropeptides. It revealed that several enzymes mediating the modification of histone and DNA are up-regulated in *R. clavigera*, while several nuclear receptors were unchanged. Of the eighteen histone methyltransferases examined, protein arginine-N-methyltransferase 1B, 3, 4, 5 and 7 were up-regulated in CSM snails. Two histone acetyl transferases, KAT8 and HAT1, are also up-regulated. Although their basal levels of gene expression were extremely low, DNA methyltransferase 1B and 3 were also up-regulated in CSM snails. Moreover, there is a possibility that ubiquitination of histone is promoted, because three E3 ubiquitin ligases are up-regulated in CSM snails. Interestingly, those up-regulations were evident in females, but not in males, which is consistent with the fact that down-regulation of neuropeptide precursors, a symptom of CSM, is more evident in females (15). These results suggest that chemical modification of DNA and histone is promoted in female CSM snails, although the histone deacetylase 8 was up-regulated in CSM snails.

Chemical modifications of DNA and histone, such as methylation and acetylation, are central to epigenesis, which enables persistent modifications of gene expression (42). Accumulating evidence has demonstrated that environmental factors such as temperature and metabolic status modify epigenetic status, which affects inheritance of traits (43). Because models of genes encoding those epigenetics-related enzymes were determined solely by the similarity to the amino acid sequences of the query, but not by any functional examination, our data is rather primitive. Nevertheless, the facts that 1) epigenetic modification of gene expression occurs during the early development of oyster embryos (44), 2) DNA methylation is crucial for gametogenesis in scallops (45) and sex determination in oysters (46), reinforce the hypothesis that epigenetics is one of the important mechanisms that regulates gene expression in mollusks. Therefore, it is likely that epigenetic regulation of gene expression mediates down-regulation of neuropeptide precursor genes in *R. clavigera*, in CSM.

### 4.5 Up-regulation of a single neuropeptide precursor in CSM snails

Other possible mechanisms for the induction of CSM include continuous up-regulation of maturation-promoting neuropeptide(s) in *R. clavigera*. In fact, we found that a precursor of MSP was up-regulated in CSM snails. MSP is a protein pheromone that is synthesized in the prostate gland and secreted into seminal fluid in mammals (47). In mollusks, MSP is synthesized in the nidamental gland of female cephalopods, and is secreted to the egg-mass as one of the gelatinous components (48). It was reported that MSP in the egg-mass induces aggressive behaviors toward a conspicuous male squid when he touches the egg-mass. In this context, MSP can be regarded as a reproduction-associated neuropeptide. To our knowledge, this is the first finding that MSP is expressed in the CNS of mollusks. However, it is unlikely that MSP is a major neuropeptide in the *Reishia* CNS, because the expression level of MSP is one of the lowest five in the CNS of *R. clavigera*. Nevertheless, the possibility remains that the locally secreted MSP at the synaptic cleft activates nearby neurons or endocrine cells, triggering sexual maturation of *R. clavigera*, as is the case for peptidergic command neuron, which triggers a series of stereo-typical behaviors for feeding in *Aplysia* CNS (49).

### 4.6 Relevance of the nuclear disaster to the development of CSM in marine snails

Because CSM is found in a site-specific fashion (in the area between 1 to 3 km south of FDNPP) and was never reported before 2017, a relationship between the nuclear disaster and CSM is highly likely. Our data clearly indicate the disturbance of gene-expression of regulatory neuropeptides in CSM snails. In line with the idea that neuropeptides play crucial roles in the regulation of reproduction in a wide variety of animal species, it is likely that malfunctioning of the regulatory neuropeptide system promotes the gonadal maturation throughout the year. The CSM in *R. clavigera* demonstrates that the nuclear disaster still has negative effects on the ecosystem in the intertidal zone, even after more than a decade has passed.

However, the environmental factor(s) that induced CSM is currently unknown. As mentioned above, CSM was induced in a new population that migrated in from an outside area, after the contamination by radioactive substances had decreased. Therefore, it is unlikely that gene-mutation by exposure to high levels of radioactive substances induced the CSM. Leakage of heavy metals from cement is assumed to be more sustainable than contamination by radioactive substances. Although toxicities of heavy metals such as copper and cadmium in gastropod mollusks have repeatedly been reported (7), these substances have negative effects on reproduction, including on gonadal maturation. Considering the fact that organotin compounds induced imposex on this marine snail through the activation of a nuclear receptor, retinoid X receptor, another possibility is that yet-identified chemicals derived from the nuclear disaster mediate the induction of CSM. Regarding this possibility, expression panels of neuropeptides in this study would enable us to construct bioassay systems to identify chemical(s) responsible for the environmental disturbance that has occurred around FDNPP.

## 5 Concluding remarks

Comprehensive expression panels of snail neuropeptide precursors made in this study will be useful for application to environmental assessment as well as to studies of marine reproductive biology. We found that gene expression of a wide range of neuropeptide precursors, such as conopressin and myomodulin, is modified in CSM snails. These precursors clearly constitute molecular markers for CSM. By monitoring the changes in expression of these neuropeptide precursors, CSM in *R. clavigera* can be rapidly predicted even before morphological changes in the gonad are clearly evident. Targeting malfunctional features of the regulatory neuropeptide system would make it possible to develop a rapid system for environmental assessment and further elucidate a mechanism of sexual adaptation of a marine species to ecological alterations.

This report has opened a door for elucidating the molecular mechanism for CSM. For achieving that goal, our comprehensive identification of 88 relevant neuropeptides would provide a molecular toolkit of bioactive materials. Taking advantage of this comprehensive dataset of gene models in CSM in snails to perform further investigations with a variety of viewpoints will be essential.

## Data availability statement

The datasets presented in this study can be found in online repositories. The names of the repository/repositories and accession number(s) can be found below: https://www.ncbi.nlm.nih.gov/geo/query/acc.cgi?acc=GSE212768, GSE212768.

## Author contributions

FM, TI, and TH contributed to conception and design of the study. TH collected the materials, and FM prepared library for RNA-Seq analysis. TI organized the database. TI and HA performed the statistical analysis. FM wrote the first draft of the manuscript. All authors contributed to the article and approved the submitted version.
