# Supplementary material for: Concomitant downregulation of neuropeptide genes in a marine snail with consecutive sexual maturation after a nuclear disaster in Japan
Source: Front Endocrinol (Lausanne). 2023 Mar 10;14:1129666. doi: 10.3389/fendo.2023.1129666 (PMC10036341; doi:10.3389/fendo.2023.1129666)
Supplement: Supplementary file 1 [file DataSheet_1.pdf]

Supplementary Material

Concomitant downregulation of neuropeptide genes in a marine snail with consecutive sexual maturation after a nuclear disaster in Japan

Fumihiro Morishita[1,3)], Toshihiro Horiguchi[2)], Hiroto Akuta[3)], Tatsuya Ueki[1,3)], Takuya Imamura [1,3,4)]

1: Program of Basic Biology, Graduate School of Integrated Sciences for Life, Hiroshima University, 1-3-1 Kagamiyama, Higashi-Hiroshima, Hiroshima 739-8526, Japan

2: Health and Environmental Risk Division, National Institute for Environmental Studies, 16-2 Onogawa, Tsukuba, Ibaraki, 305-8506 Japan

3: Department of Biological Science, Faculty of Science, Hiroshima University, 1-3-1 Kagamiyama, Higashi-Hiroshima, Hiroshima 739-8526, Japan

4: Program of Biomedical Science, Graduate School of Integrated Sciences for Life, Hiroshima University, 1-3-1 Kagamiyama, Higashi-Hiroshima, Hiroshima 739-8526, Japan

Correspondence to: Fumihiro Morishita, Ph. D.
Program of Basic Biology
Graduate School of Integrated Sciences for Life
Hiroshima University
1-3-1 Kagamiyama, Higashi-Hiroshima, Hiroshima 739-8526, Japan
e-mail: fumi425@hiroshima-u.ac.jp
Phone & facsimile : +81-82-424-7439

Co-corresponding author : Takuya Imamura, Ph. D.
Program of Medical Sciences
Graduate School of Integrated Sciences for Life
Hiroshima University
1-3-1 Kagamiyama, Higashi-Hiroshima, Hiroshima 739-8526, Japan
e-mail: timamura@hiroshima-u.ac.jp
Phone : +81-82-424-7438
Facsimile : +81-82-424-7439

1 Supplementary Figures and Tables

For more information on Supplementary Material and for details on the different file types accepted, please see here.

1.1 Supplementary Figures

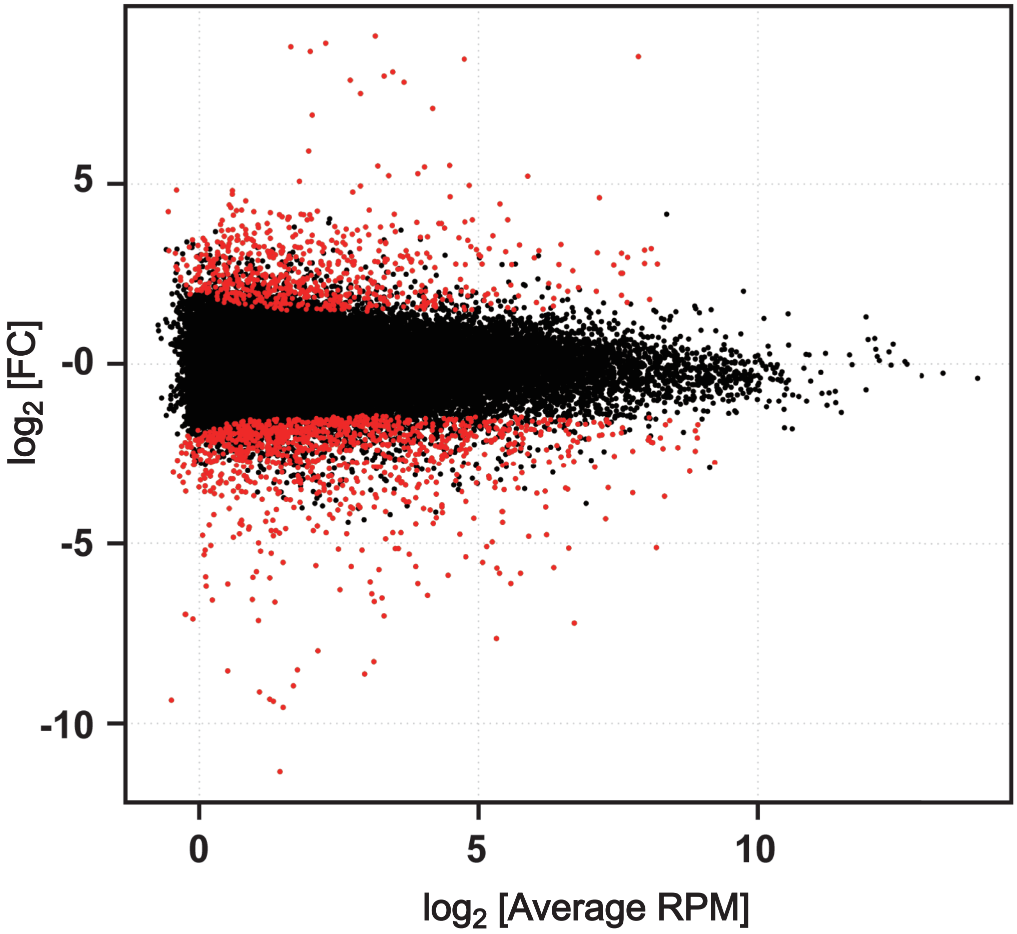

Figure S1. MA-plot of transcript models assembled by RNA-seq analysis on CNS of female *R. clavigera.*

Differentially expressed clusters between the normal- and CSM-female snails were shown by the MA-plot. DEGs between normal and CSM snails with false discovery rate (FDR) below 0.05 are represented by red dots. RPM: reads per million mapped reads, FC: fold change.

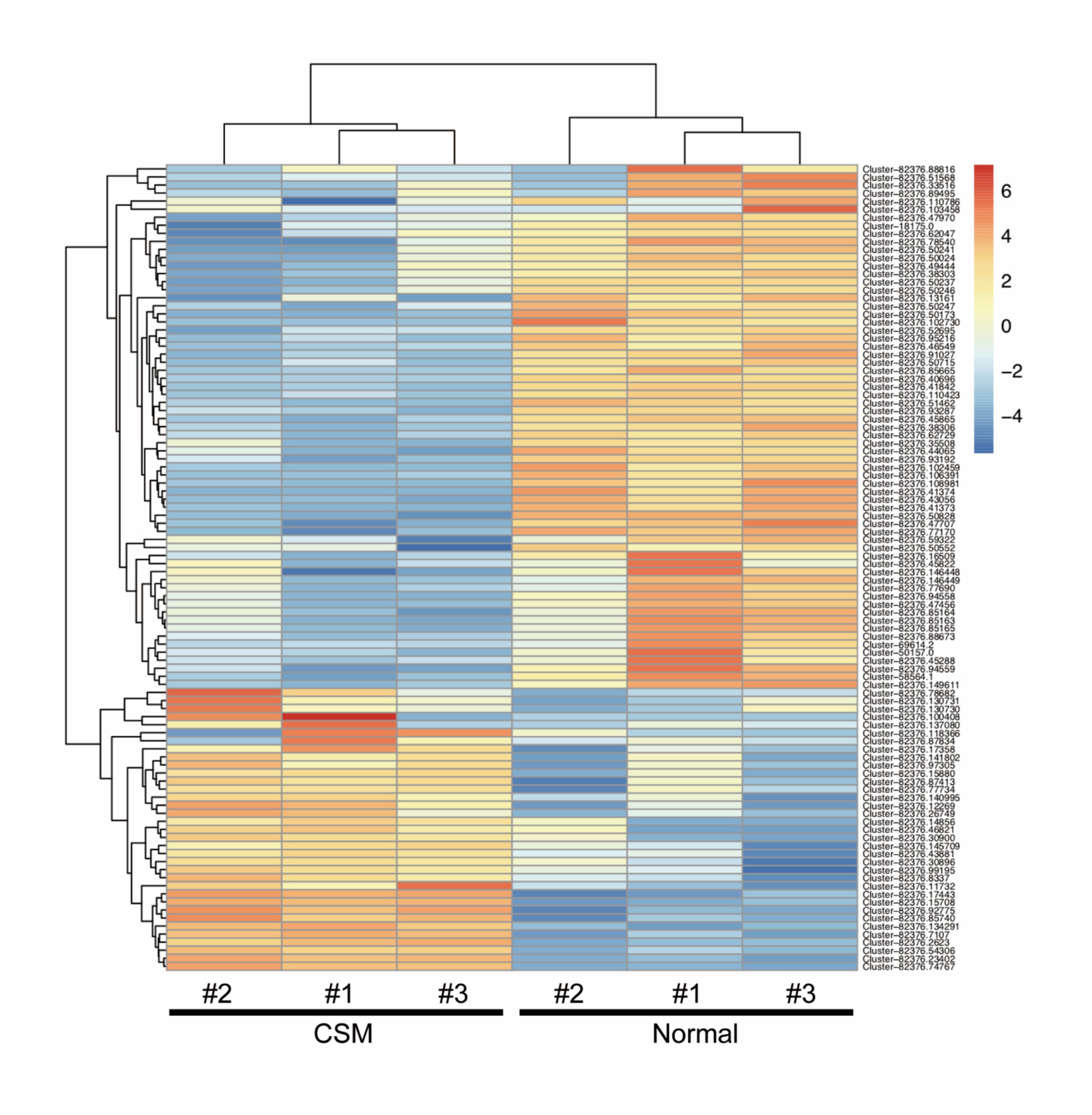

Figure S2. Heatmap of top 100 of DEGs in normal and CSM female snails.

Heatmap of top 100 DEGs was made for the representative samples of normal and CSM female snails. Fold changes in each DEG were calculated using the $\log_2$ value relative to average RPM, and represented by color.

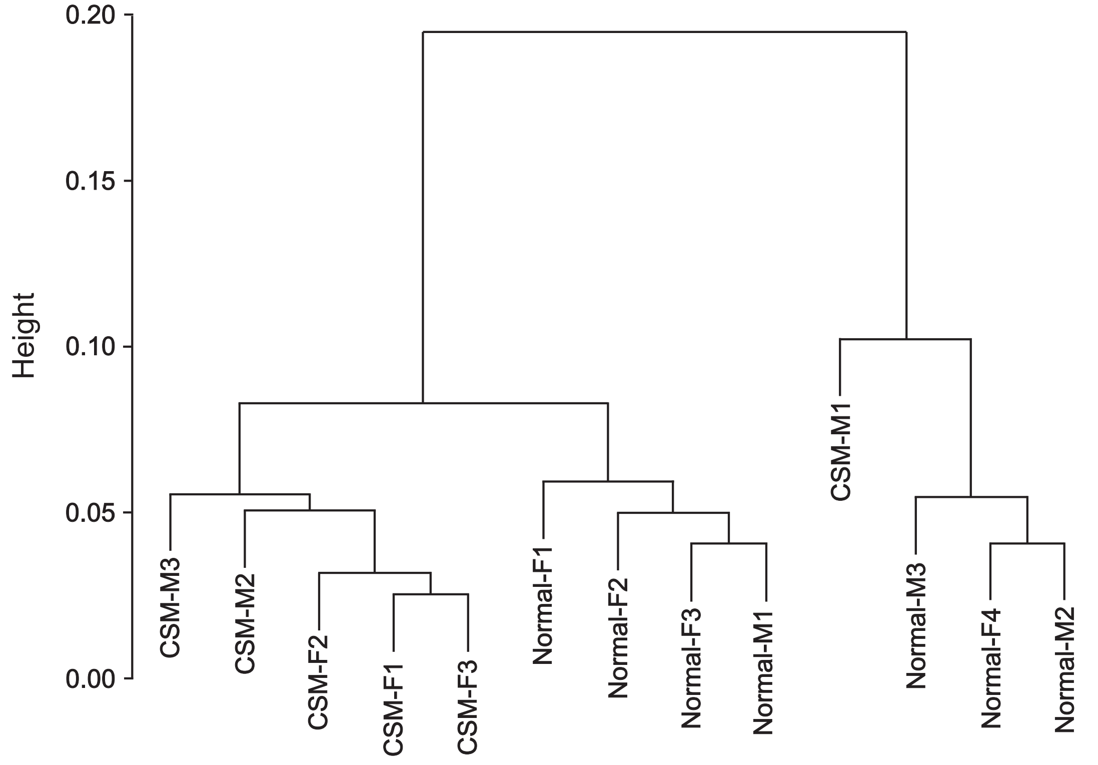

Figure S3. Clustering diagram of cDNA libraries of *Reishia* ganglia used for the RNA-seq analysis.

Clustering diagram of cDNA libraries was made on 13 libraries of *Reishia* ganglia of the normal and CSM snails. F: female, M: male.

>Achatin-I
MTTSSRYLLALAVVIATVTLLLADDMDFADDVSFPFADDDVTLGDLFFTKRGFGNKRGFGDKRGFADKRGFGNKRGFADKRGMGDKRGRLYSPASVIAAL
FRSYYHGQPLAGDIALKRLLQKQGFWQ*

>*Achatina* cardioexcitatory peptide (ACEP)
MQRVTEIVLIACVMMVMTSYMQQTEAAPSWRPQGRFGKRGGAPFLDVAKSTAAALNGEIEIPVEVMASGERGLFLRVGVKPCTITGVDGVAPCSG*

>Adipokinetic hormone (AKH)
MTLRSHVMCVVALLCLWLVTCPVRSQIHFSHSWGTGKRSHQGQGGYAGAASAPFFTVDSSGAGHEGDVCWRGVDVRIMAQLIHLIRREVDRYSRCQMDDI
IAAMPAGVNRVSDDALKH*

>Allatostatin-A1
MARQTVTLWIERFVVVLCLWGWSSCLADFDKKEETGIQQRLYMLPSTSLTRTRDYLHSAPEASRIPDESGESKHKSIDQPGRRYLDLHNFYEGLDKRVDR
HNFNFGLGKRRGDQNAFRFGLGKRQIDSNSFGFGLGKRQVDRNNFSFGLGKRKMDSANFKVGLGKRGVDRHNFNFGLGKRRGDQNAFRFGLGKRQIDSNS
FGFGLGKRQVDRNNFSFGLGKRKMDSANFKVGLGKRGVDRHNFNFGLGKRRGDQNAFRFGLGKRQIDSNSFGFGLGKRQVDRNNFSFGLGKRKMDSANFK
VGLGKRGVDRHNFNFGLGKRRGDQNAFRFGLGKREIDRHSFSFGLGKRKVDHNNFGFGLGKRKVDHNSFGLGKRKMDDNHFRFGLGKRRMDGNNFMFGLG
KRRMDSNNFMFGLGKRRMDSNNFMFGLGKRRMDSNNFMFGLGKRRMDSNNFMFGLGKRNVDRNNFNFGLGKRTMTIPDLSVGLGRGLTPSMRYRLSQGKR
RLDHTNFLVGLGKRDVQEWPRMWTSYLTEGHLQPELHRGSGVPWSKVSETLHSYDARDSDPTEFGPPVTPSLPDQRATSQPWLKKSPAWELGSSVDKKSV
YGL*

>Allatostatin-A2
MLSTSPARLFFLLVCACLCLSCDSVPFRTDDGKSFTFRVYTNGPLVSHTPPAILSSLEARSGTDDSSWSPALSRDSLIPLQDPDFRYAGLDKRSVDFRHF
RAGPLKKRRLDRINFHVGLGKRQADLDSLGHDLQQPAKDGVHVVGFERSLQELFRLLDAFRRQSESEEYSGREQQGVDFDEDDYKDGLAKKRMDTLNFRV
GLGKREAGPRRYILNKNYLDSLDLISSLDNKNIDLQPSGEEKRTIDRTNFRFGLGKRNVDRHSFMFGLGKRQIDRNSFRFGLGKRTIDQAGFRFGLGKRR
VDGNNFRFGLGKREIHENDFEHGASAHGEEGHGFRVGLVKRSVDQHTPHREQDPDNTDVNNTSSAQHDHPDGNQNPKPTSAKRPMDSNNFGFGLGKRTVD
RTNFGFGLGKRKVDHNNFGFGLGKRGVDHNNFGFGLGKRGVDRNNFGFGLGKRGVDHNNFGFGLGKRNVDRNNFGFGLGKRGVNNFGFGLGKRSVDRNNF
GFGLGKRKVDYNNFGFGLGKRKVDYNNFGFGLGKRKVDGSQQLWIWVGKKGSRSQQLWIWVRKTEC*

>APGWamide-1
MNLFHQMFSVSLLAQVVLLSYHTAVLGDEEKPSDLVQGSAADSQMQTLRLKRAPGWGKRFSELDPEVDASEEEDSDFVGAEACPTCAEDVDGADLDMDKR
APGWGKRAPGWGKRAPGWGKRAPGWGKRAPGWGKRAPGWGKRAPGWGKRAEDLSSLDGLLDGEQFELVGKRAPGWGKRAPGWGKRAPGWGKRAPGWGKRA
PGWGKRAPGWGKRAYGDSS*

>APGWamide-2
MTGRMLKQLFVTLILHHLFLDCGQSATAEQTEEKESGALSSFRLPTDTDSAPVPVLRQKRAPGWGKRSPVDVDNFDSEDMLNDLDDDDDADSLEVKRAPG
WGKREFETGKRAPGWGKRAPGWGKRAPGWGKRAPGWGKRAPGWGKRAPGWGKRAPGWGKRSMLADDTEVKRAPGWGKRAPGWGKRAPGWGKRAPGWGKRA
PGWGKRSLEVEGDKCSLWSEELEFFINNAVQTEARLQALCGYNTATLHHLSRK*

>Buccalin
MLTKTFKLTHLFVCPFLLLITTTTLSHADAPVSVEAEQQKSLTDSTYDKTAGMDTNALLSSLDKRGEKPGMDRLAFFGRLGKRSKEDSDQLDKRGMDEYA
FMGRLGKRDSKASGQENQDEEPTHLSTGRTKRGLDDFLSTSNLEKRPMDNRMFGPQLGKRRMDNMMFGPQLGKRERIDNMMFGPQLGKRERMDNMMFGPQ
LGKRRMDNMMFGPQLGKRRMDNMMFGPQLGKRRMDNMMFGPQLGKRRMDNMMFGPQLGKRERMDNMMFGPQLGKRERMDNMMFGPQLGKRRMDNMMFGPQ
LGKRERIDGRMFGPQLGKRERMDSMMFGPQLGKRERMDSRMFGPQLGKRERMDSMMFGPQLGKRERMDQFMFGSRLGKRQADWEDDSTEKEVADNDESS*

>Cerebrin
MLSISKVTLLVTLMTLMVTWVSHETQATPLAPYSAGLEDRARQDIITLAARIIKMAMSGRSMEAGASKRNGGTLDTLYNLPNLNDIGRR*

>Cholecystokinin (CCK) -1
MTHALRLVLTTATALLLSAYVTTAVAVPAARADRVQGGLTQLAKLINRLKHLDNTDNNNNDDDDDDDNAWAALSGKDEEDLSSHSTGPAVSPSVDKLTAL
RQGFLGFNKRQGTWSYDYGKRYYGDYGIGGGRFGRDVDHVDIADTSDVTL*

>Cholecystokinin (CCK) -2
MEQSQHVLVTLTALLLCTCAVASLPAATQPEELQGILSHLFTNVVSKLKTSHTTHEASPNAWPIADSEGNHQQALDTGSASVSSNINKLSALKHGFLGFN
KRQGYYDYGLGGGRFGKRYYGDYGIGGGRFGRDVDHVDIADTNDSAQ*

>Clionin-1
MVGTHSLAMVFALTLALAAASAVLPIIPLKDMAPEHACFFICDICLAEGPKEDLLKCANEVCGRILPGATCGPVLDLNMLWLGQKCRHLDFVLNLMLGNT
PMKKREQS*

>Clionin-2
MMQVQNLALVLALTLTVAYAAVMPVIPLKQMAPEHACYFMCEICLEDAPEEDLLHCATQVCGRIPTGATCGPVLDVGMLWLGHRCRHFSQLMHSMSSTET
EKH*

Figure S4

Amino-acid sequences predicted by translating nucleotide sequences of transcript models assembled using RNA-Seq data are indicated with predicted N-terminal signal peptide, predicted processing sites, and predicted amide donors. N-terminal signal peptides are highlighted with light blue. Predicted processing sites and Gly residues for amide-donner are highlighted with red and dark blue, respectively. Predicted mature peptides are highlighted with yellow. Unique peptides in the precursor are highlighted with magenta. Asterisk represent amino acid sequence terminated by stop codon, while hyphens represent undetermined N- or C-termini of a precursor.

>Conopressin
MMKCSAVATSRVWWTLCLVLLQVGAVHCCFIRNCPRGGKRALEPMGAARQCMSCGPGGVGQCVGPAICCGPDIGCLVGTPEAEVCQKENESSSPCVVSGR
HCGMDNTGNCVADGLCCVEDACSFNSLCKSSVAEEEDPKSTRHELLSLIRRLLINRQYD*

>Crustacean cardioactive peptide (CCAP)
MMPRGHVLYFILLPVLAQVAADETDEQIMSEVISGEQDRASSRANALMRSPPHIPLPDSLLPPALLLQLLQSSDIPHPAPLSSSSSSESLSSSLHSESSD
EVDDNGEVTKRVFCNGFTGCGGRHRDRSRRQERYGKRLIPLLVKRPFCNSFGCYNGKRSSALPLVNPGENYRARVLAHMLAASKTARHQPQEGMGVERAP
MTGGVLGKRLFCNGYGGCRGGKRSLFSPWVNKMNGVVGEGRR*

>Egg-laying hormone (ELH)
MHCVFTTPTATTTTTTTLLLLFLLLGLFSAHGLPTASRSGLATHLEAGEDLAQLLSGPSMAKTLTKRDLSINRDLQSLADMLLSKERSRIAANNRNRAFL
RELGKRSNDFDLNEESDEGLVLPSLLEPSEEWLYRW*

>Elevenin-1L
MAASQRMFQLLIVSLLLMTSAARRRIDCTRFVYAPGCRGVSAKRGASPLSVQGSTELNDALTDAMLRAAESKEERDTEKNWPQWSRLSRVLRLLSQSKRG
AERLEQLLPLQ*

>Elevenin-1S
MAASQRMFQLLIVSLLLMTCVASQFTDCTTSPWVCHGVSAKRGASPLSVQASTKLNDALTEEVDSLGEGKILL*

>Elevenin-2
MVPSTSQSRMVVMVMMVALLLAVPHVSSWRIDCTRFVFAPRCRGVAAKRGDNSLSAKQSEALVGDDNTQSLQGSSVEEAQRRDPEAFLQLLMALAPSMPR
SMPLTQRRKEVMEELLSS*

>FFamide-1
MMKGRAVCLVLALCLALTAAHTPGTDRRSLSRLVGQQPLLFGRRGINPNMNSLFFGKRASYPLMPPASLEDLSAACNVLLSTYRQMAMRTDSDS*

>FFamide-2
MSARLVCLVLALCLCVAASHTQGTARRSAINRLVGQQPLLFGRRGINPNMNSLFFGKRNSAGEQYSRAGLKSAWPMLLPPAD*

>FLRFamide/FMRFamide
MLTTKDMPRPTGWLCLAAVTMTTLAMMADVTSAYRSICSKSANKKICYYELSKRFLRFGRALTDDHFLRFGRNMDKRFLRFGRPDADSSLDDMLRLAIAH
AQSEDTPLYIRKRRSTAEAPQEAAQAASADHPEGVAKREADNSDLPIEDDKRFMRFGRSGDDSDDLEENKRFMRFGKRFMRFGRGDEEDGMQEDKRFMRF
GKRFMRFGRGAEDEDDSLSQDKRFMRFGKRFMRFGRNSDSGGYEDEEDDKDKRFMRFGKRFMRFGRSGLDEEEPSEEKRFMRFGKRFMRFGRDPMEADKR
FMRFGRSGQDADDAKDKRFMRFGKRFMRFGKSGKAAESKSEQASS*

>FMRG
MKKKTRMASSSWPLLLSGLVLFCTSGIVEGDVPSPEKPEPQPKNTSAPKPQDSAASAGPKLTAHGDPEKLLQDDLQLPPSQAKLHSTAEDGQTEKRFIKF
GDSFFANLGPSGAGHLDGSGVGSQGARAALDKRFMRGVDAYIRAGKRDSGYVISEPEKKFMRGVDAYIHAGNKDTGNMPEKKFMRGVDAYIKAGKRDHVS
VPQKKFMRGVDAYIQAAKKDTGNIPEKKFMRGIDTYIQAASTQPGFSLPEKNL*

>FRFamide
MESSQLAQVLVVLMSCLLLAVVTCDTSHDTAMEDAAPSQPQKRSVADLPAEDLPYTAYYPDLEPLDKRSSLFRFGKRGSLFRFGKRGSLFRFGKRSSLFR
FGKRGSLFRFGKRDGGDLEDAWVLYPEADLPVEDLKRDVKSFHWGRETEE*

>FRYamide
MKTSTSLCLAFLMVCLLCGASLAEEPLLDLQSYCVLECSLGESTFCDCDDLADRGLTSAFKRGGAFPFRYGKRDLVEKRQMPFRYGKRSLESSHPWWARN
AAANFAAALSDHQFARSLIKRQFRYGKRSVPSPAAAFESQDAGSATKRQFRYGKRSAPNSVVDLLGYNLPKASALTASSSEAEK*

>Fulicin
MQGIGQLLVSTLLLLLPLLSVLAHKTDDSPSHLNKRSLHEVSSEETSWQAPLSSDDVTAAKRFNEFVGKRAQSLLSTSKASEMVNKRSPYELLYNRILTK
RSAGDAPAQIVKRSSQYEFLGKRPLSLPSHKRAVLDYLAKRVLLPYEFVGKRIFGSAWERVSANAIEGDALIKDVTDILNKRDSVNHQNIPEVRNKRYAE
WLGKRGGPIADQLLRDLVNKRISVMMRNRLQSSAPEFIGRREEDSLPQDEVQKRYTEFIGK*

>FVRIamide-1
MNLIIIPAVTSLVLLLSTLSRAAPNDVNKASASDVSSDVSDDSELAKRISSFVRIGRPSSFVRIGRASHFVRIGRPGNFVRIGRGYPSNTDDMGYDSQDL
GYGADKRSSKFVRIGKGSSFVRIGKSMVPQDKRMSSFVRIGKGYPFGDVYDDDLNKRVSSSVRIGRTPTFALLRSGAMDNGEDMDMAKA*

>FVRIamide-2
MQMNTFLPLLSLFSPLILSVLVSAAPSDVSKQSDVTAPDSQYDDVSDDTDLAKRLSSFVRIGRPNSFVRIGRGSHFVRIGRPGNFVRIGRGYEGDDVEDA
VDTEGESLGADKRASRFVRIGKASRFVRIGKSGQIDPEIKRMSSFVRIGKADPYAYLDSDDSNKRASSFVRIGRIPSSAFVRIGRNSGEGAGEDGDFGTF
NRFARMGQSSFVRIGKREADPQKLAEKVESDNLKH*

Figure S4 (continued)

>FXXFamide

MGLLCSVRCRQLMMMMTLILMISFSSLIFTIQAAEDGVTTTEPKPADSKPQQEQTAKQSDDDTTGDKSKRGFDPIGGHSPFAKFGKREDENSNIQVQERK
FDRIAGSSAFGRFGKREDTENNEADKRFDRISGVSGFGRFGKREGAEASEKELKAQNSEEGQGDGTMDKRRFDRIAGATGFGAFGKREQGESDKRFDRIS
GLSSFGTFGKREEEEGKDKRFDRISGLSSFGSFGKREGDKDKRFDRISGLSSFGSFGKREEEGKDKRFDRISGLSSFGTFGKRGEEKDKRFDRISGLSSF
GSFGKREEEDKDKRFDRISGLSSFGTFGKREDGEDQKRFDRISGLSSFGTFGKREDGEDQKRFDRISGLSSFGTFGKRGDGEDQKRFDRISGLSSFGSFG
KRGENKNKRFDRISGLSSFGTFGKREDGEDQKRFDRISGLSSFGSFGKREDEEKNKRFDRISGLSSFGSFGKREEEDKDKRFDRISGLSSFGSFGKRDDE
KDKRFDRISGLSSFGTFGKREADSDLNKRGFDRISSASGFAKFGKRQSEMLADEPAVDSEKRGFDRISGMSSFAKFGKRGFDRIGGMSSFSSFGKRKFDR
IGGMSNFAQFGKRYDGSPDNSEAFEDQKRGFDRIGGMSGFSSFGKRDLMEDSQDQEKRFDRIASVSSFGKFGKRPADGSDDDLNKRRFDAISSSGFGRFG
KRDSTDVSGPDKRNFDRIAGNSRFTSFGKREDAGASGEISDNSKRFDRIGQSSFRSFSKRNSSVEASEDTEAKDSPRPVTPPPTPTSKSQQSEHSKHISK
RDLRSLMDNDKRRFDSIAPSSGLAVFGKRGLDSIASYGKFGGFGKRRLDSIASYGKFGGFGKRGLDSIASYSSKFGGFGKRGLDSIASYSSKFGGFGKRG
MDSIASYSSQFGGFGKRGLDSIASYSSKFGGFGKRGLDSIASYSSQFGGFGKRGIDSINSYSKFGGFGKRGMDSISSCSRFGGFGKRRLDSIAPSSTFGS
FGKRDGDNIWDDLATINDRLHLEGDSLTPEDVQHLMYLLTGSVPQEITNEGGEASDDDQAWQDLEEDIQKRSHDPIGHYGRFGRFGKRGGGDEDEEEKVA
FLPLSSLWREDVSKLCGPLVADLDDKESMSKRAFDRIATSGFARFGRVAWTPSAPAVLSKSSSSSSVPSPAIRMDSSYLPFCEVALRLQQYLDTFHNHLT
ATNDSARHKREFDPIAYGQFGKWGKKRRNAIPPVPRQPVPS*

>GDPFLRFamide

MWCTLRCLWLLALVVVLSALLHGSAADDDFCGRPANMDLRWWNNNYKRYLRFGRGDPFLRFGRSKPESWPFNRFQKREEDPDSLEKYLQNGGYLRFGRSV
PMLAFAIRKRRDVRSAPSSAELHRVARNVKDGGFLRFGRQQTPSNAGQDPCSQQVKGGSAKKSDVDLPFDNKHYLHFGKRNYLRVARNVKDGGFLRFGRQ
QTPSNAGQDPCSQQVKGESAKKSDVDLPFGNKHYLHFGKRNYLRFGRTYPGGLSPLWKTGCRNGLDVDELDMIVFFSSFLSSFFLNQEVSL*

>GGKVamide

MLTRAWNLAAVLLFGSVLLVKSSRGRSMAEKDTPGLPSHGEMPIGISEVIRRANGGKVGRTHMIDSQTGEELILDGMDMFLTMEQYARLYGHHFDHTNHE
QVETTTTAPEILDVISRIGDVINSTTATAKKEEK*

>Glycoprotein hormone (GpH)-α

MSSGSKSVRTAHSKVVVCIMVAWLCAVLCGNCPQCEANSDSSSQSWRAPGCHLVGHTRRVKMHGCLTFQVTTNACRGFCLSFAYPTPESTRMYHPEYIFT
SRASCCNIIDTVDIPVQVRCGDVIREVVFKSARSCGCSICRRG*

>Glycoprotein hormone (GpH)-β

MFYAMPKLTGDKLTRLMRRLQHLTVLLLLLLLGLGLSPSPSPSSSSSPSILCGASASSIVNPRTTLKCHVRSYTFRASKPPIINKNGDLVTCEGDVHVKS
CWGRCDSSEIGDFKMPFKVSHHPVCTYTGRVSRTVRLSDCAGYPDPTVQVFDAAGCACRLCNSDFTSCENLNG*

>Gonadotropin-releasing hormone (GnRH)

MSPSRTVIPSAAFLLVVVVAVLLDHLGSAHGQNYHYSNGWHPGKRGDVEDSRQTAMYQTLTGDHRCKVRQNILQIINHLITEEIARLGRSCSSQDNFDFL
RSLMEPSRAAPARRRRRKGQREEEGGEGGASVMQW*

>GnRH-related peptide

MLQAPCRLSFLSLLALLPWTLLLVSATKAQHYHFSNGWHPGKRDSLGEIDTRSYHYSNGWNPGKRETGDRETRTYHYSNGWNPGKRESGDRELRNYHYSN
WWNAGKREEDSVERKARNYHYSNGWNAGKRSEALERETRNYHYSNGWNPGKRSDTLQREARNYHYSNGWNAGKRQDPVTVQTRNYHYSNGWNAGKRQDPV
TIQTRNYHYSNGWNAGKRQDPVTIQTRNYHYSNGWNAGKRQDPVTIQTRNYHYSNGWNAGKRADDLEREIRNYHYSNGWNAGKRADTMKREIRNYHYSNG
WNPGKRGEGMKRETRNYHYSNGWNAGKRAEALERETRNYHYSNGWNAGKRAEAGERETRNYHYSNGWNAGKRQDPVTIQTRNYHYSNGWNAGKRGEALER
ETRNYHYSNGWNAGKRGEPGERETRNYHYSNGWNAGKRTDTLERETRSYHYSNGWNPGKREAGDRVSRSYHYSNGWNPGKREEAKNLSDLENIDDDTSIK
AAAAAAAGEDEEEDCCGQDLEVLALVKQLEMPPSSSVDAKTSSEEDSEKE*

>Helicostatin-related peptide

MAIQKRFCLLNWTVLFLVTLFSSQNLTEAQDLKATASGDEEIIRPSQASDLCKSDESVANLCGACAALPGMVSFPVELCCNDVDVMAVCGDCLTDPSTCL
LDIITLQNVISAAVNSDPELDILEPTDALSTDYYYDDDDYLDDDADLPELEKTKRFGTLYLGKRPLYVSQPPPALSDVIKRDDIDEVMEPDSDADDVEKR
WGTLGLGSGNHRRYARWGSLGIGKRWGSLGIGNGNRRSYYGFGKRWGSLGIGNGKRLDAPEVDKKWGTLGIGKRWGSLGIGKRWGSLGIGKRWGSLGIGK
RWGSLGIGKRWGSLGIGKRWGSLGIGKRWGSLGIGKRWGSLGIGNWKRWGSLGLSSRYRGKRWGTLGFGKRDTASGADDSNNPDTPVEENKRWGVLGLGS
RIKWGPSLRGKRWGSLGLSSWRKRLGRLELEGKRWGSLGIGKRNEILTALDDNANYLNTLTDSSAEDKRWGTLSLGKRGNAAALNMEDGVGPDSSEEKRW
GTLGIGKRWGSLGGFGGFKRWGYIGLGHRNPYYSHRPRSIGKRSAGVGEPIDKRSGSEPQDKNEDEEKDADAKSKKWGSLGIGKKWGSLGIGKKWGSLGI
GKKWGSLGIGKKWGSLGIGKKWGSLGIGKKWGTLGLGKKRVNPGTKEKQSSPHVTTEQMKKDASE*

>*Helix* command-neuron specific (HCS) -2

MKSLLRILEEMLALWLVCLLAGSSCAVFYTQSNNNDFPRLGRRSVFTASADGVYPRIGRSSATLSHDEAEDSLFDTSFQSSHHKRGIFTAGGPGFPRIGR
AQRKQSRGGGELSPHAVSVVTSVESPDVERGVGAEDEETRFEYPLGLMFFEFDKDGDKQLSREEFVQGMERARHDGTLFR*

>Insulin-like peptide 1A

MGAVRAAFQRIAMTEVTPVVLALLCMLGQTTGQTFCGVDSRPHPKGLCGSMLLRAHRNLCFLLSADYPNIFSRSIQKRALKNIDDLPLRAYAEMDLQGPE
YTENGPSGPVPNSANIRKLIMSLFPRVQGLLPDLATGTSQMGQGPRPMAGRLQAKRAEDMAPHVQKRGMVCDCCYNVCQASTLAQYCP*

>Insulin-like peptide 1B

MAVPGPSYPPVLLPLLLSLLALHLNCAVYGSYEHTCTLDTRSRGAHPNGICGKNLAQIVSVLCAPRGYVSNWFTKRSAPNRPDEGFVSKNLRGILLNKKE
ALSYLQKREPRAARRTFGSQGITCECCYNRCSYYELLQYCN*

>Insulin-like peptide 2

MAAAWLWSPLAVLGLLLQVCLVQAGYEHSCYVDDRVPHHRGICGASLSNTLGQLCRMMDDGRRKRVSTALASDADEAQQMRLRDHLLSKRQAMSFLDKQQ
QQQQQLQQQQQQQLGRQQQQGPASVLSLVGMPPLLRKRAKTQGITCECCFNHCSVRELLQYC*

Figure S4 (continued)

>Leucokinin
MNMARAVGKLVCTICLAAVFVSFVQSSTSEAEASKHGGNNSGVQQDSESHTSDSDRDSKQAEEAERTAQGREARGDSLAGFNKKTNEHTPKDSSMAANNP
GHKEHHFQSAPAAQSSDTSNSVIYPPDSSSEEPSSEDYMSVDEDSYQSEDVKRAPFSVWAGKRSPMEGYTEVDMESPFAAEDAEGYGEESQNMNEWKRAP
FSVWAGKRSPDVQDDDEFSKRKAFSVWAGKRSQDVDSESISKRAPFSVWAGKRSEDVELEGASKRAPFSVWAGKRSDDADSETPSKRAPFSVWAGKRSDS
TESQGTSKRAPFSVWAGKRSENSESVESSKRAPFSVWAGKRSDVNTLEQSAKRAPFSVWAGKRSDDDASLDFLKRAPFSVWAGKRSDDNSIDSAKRAAFS
VWAGKRSDNDHSTDSSKRAAFSVWAGKRADDSSVDSVKRAAFSVWAGKRYDGSSAPLRSSKRAPFSVWAGKRSEDNDSAEPWKRAPFSVWAGKRSENPDS
AVQSKRAPFSVWAGKRSDGSDAADSSKRAPFSVWAGKRSDSPDSDVPSDKRAPFSVWAGKRSVDPKSINPSKRGPFSVWAGRRRRSANTRTAASARPGKR
NLPARAFSVWAGKRSLEESDQKRTERSVSMTKRHTVGSASPVGLSTDTADPEMHSIHKRAAFNAWGGKRAAFSSWAGKRAFFPFWRTTTMPWFLWKPKRT
PFSAWAGKRSNSYDVEDDDDDSKRAAFSSWAGKRSGSMDSKDSLMNGLIPQDWLKSEDHENENLMNSKREAFSAWAGKRSGQSQLGESGLNGQMPLFKAD
MIPKGLYTADGLDTSKLEYPLSNDEDSSILDKFLLEHTLFSPYSQSDLDTDLPEESTSESMTLPSKRMLHLGFSSIGSWYQRRGQEQRARKLGQYLRRAM
SQSLDKRRFSSWAGKRSAMESPQFWPETGR*

>Maturation-associated peptide (MAP)-1A
MHMQAVIGLLLSMITLLTRGRLSDRRESPRSKVLRAILLQERDYEAMSKRYDTRMAPEPLRFECCPSVSSRIRPLGGLSREGKLLQLFRSDRVVQQLYET
RCRPDVVNKPCQMVDPLIKRFSHCVQKYSYAYAFVKDFNMTQDYRLDYVRVRSGCSCEVDYSLPETGEYH*

>Maturation-associated peptide (MAP)-1B
MRMHTALGLLLSMVTVLTRGRVDKKDSAGSRLLRALLLHGREPHDLLSKRGEASTVNVPVHLECCPSETRPIQPRGGVSREGKLLELYRDH*

>Maturation-associated peptide (MAP)-2A
MPCLTSPPISRCSLPLLPLLLSFLLLLLLLVADSPCQAKTRRDFDVPAFCREKCPIGMGGNLCQCNGFHFPGKRESPFLKMARHMTHEEAGAARARRDMA
DLFDVEDKKW*

>Maturation-associated peptide (MAP)-2B
MPSQSSCPPCSLFSLPLLSLFLPLLLLLVLTSAPAAQAMDITKLCQARCYYGRGGNLCNCNAFHFAGKRDGEGETLRLPSSPLLELALQGMKHVEAHRAP
EGVDTSPSLSESRLASRGRVGERGDEQGRREVGRLFDAEERERTAAEVVNRLTSLIRSALDERLIDTYDGSRKRNQGSLPYEEADFVY*

>Maturation-associated peptide (MAP)-2C
MFTTKQHSSSSSSISTLHHMLLLLLWLLLLLHSSHTLNVEQLCEIRCNIGRGGVLCKCNAFHFAGKRSDGASRYFDDLAATFRERKYKNPNGGVTISVSG
SVRPFGDAEEKEGSGDVRMGRREDREGVMAKGLFGERGEGRATVESLSSLLKNVLSNQRLVSRSKNNKNAAQTWRESSFPFTADDVEY*

>Maturation-associated peptide (MAP)-3
MVKRKDLLKVLFVYLCLMHVSPGHPCGRNPISRFQPLTKMLGRDINSLLNLLRTSNPTLYQQVQDEWRRYADCVGLVDTGYFKRSMDQGPTSQLLSQGHQ
GRGQQMLDAGLPEGFGMITLGRSRRAERLLRLMRSVAAAAAAGGRRAGDGRREVVGDVGDVEEEEV*

>Maturation-associated peptide (MAP)-5A
MIVLRSVPSAFLLVTAIFISHTGTTVARTLGLGNKEFADMLHKRNSWWSKKSVDASPDFTPILPEAEIDASDLAEENSDAYTKLFGCYTTSCVPSFVACA
TRSRTTSSFEMCKLSHRACAVRCWDFSTSGESPMK*

>Maturation-associated peptide (MAP)-5B
MAALRLVPFAVLVAAVTLLSHTGVTSARSAALQRGSLTQLLSKRNSWWSKKSTDASSDFASIVPEASLDAQDLVENSSNANTELFLCYMSSCVPSFTACA
ARSAANAFNVCQLHHRQCALRCLQMVEGDASTLK*

>Maturation-associated peptide (MAP)-6A
MTKVSLLSAIFLLLSFVMASVTSFRFKSNGPREFGQTDFYNGKYQAWMGKRGLGQSSSWGFGRPVGDRNRYFNWKDFMANAMVKRQLSFGPRSFGNYRSY
FTDGLQDWKNTFRGTRKRDVMAEDDVMI*

>Maturation-associated peptide (MAP)-6B
MDKCVSFYVTIVVMAVAIATVTSFGGNEENEGGFGGDFFNRNYQSWLDKRGLGQTYGGFGSPLGSENRFFNWKDYLANGISKRQLNYGPRSFGNFQKYFT
DGLQDWKNTYTGIKKREAVAGKDHTETGA*

>Maturation-associated peptide (MAP)-8
MTSAAFLAITVIFGVLSFPEGTQSFIRKTRAFKNDNFQRLPYKRSGHNFEGGDPKLPLANFEPSWLFNEGDITVEEMADRLMRNRHLALDFVTSFMDTDG
DRLISASEILPPSERRRR*

>Microseminoprotein (MSP)
MASIFVHCLLALCLVSTVMADCYHDRLRYQRNSNGQMERFCLTSEVKPQKIAVGKTMNTDDCYECACSHHGLRCCGFGVKAGKNAQRVPAPPGCMLVVDG
CYYYFLDPVTQQC*

>Myomodulin
MKLILSFTLCVLLPLTQVYGDDTSPDQTQNTGEAVSSDHEDLSRAKRALDYRRLARGIQMLRLGKRQLPLLRLGKREYPNDDLLLQNLLSSLDSQYYDED
YQSAEEPLHARYRRSADPSQETMPAGTLLADSTQGSSSSAVKRSVNDLDDVGAEDSYLGDEDNAMEKRPMGMLRLGKRPVGMLRLGKRPVGMLRLGKRPV
GMLRLGKRPVGMLRLGKRPVGMLRLGKRPVGMLRLGKRLTDLDSSDEEFEDSENVFDPDMNEEEDFSVENSPDGLLPQFQSDMPVSELEKRPVGMLRLGK
RPVGMLRLGKRPVGMLRLGKRPVGMLRLGKRPVGMLRLGKRQLPLLRLGKRAAE*

Figure S4 (continued)

>*Mytilus* inhitotory peptide (MIP)-1

MQGKGQLLVSTLLLFLFNVFAQNAGAHASGRSDSDAGITSSSPASSAVGISSNSAAAFSIPAAMNEGLDAMQTNLSPSFATGDADTPLLFLNDTNKQPLYR
PSSSRVHSSPPELGNLGLKDDRNVHSEKQTERDHGHYHRHFRRGRRGGYNVPYFVGKRFLDDMPDVQQDFEKRDSLDESLLKTLQDQQLLLSASRQAGSSM
STDKRYTPLFIGKRSTKHHPQEHRVRRQAYGAPYFVGKRTLEESEDLEQEELQKRSIPNDSPTDNLQDLQDFEILDLNEETPSDVLDKRHAPLFIGKRRAP
FFIGKRWVPYFVGRRQAPYFIGKRTDLTASRRAAPLFIGRRRHAPLFIGKRHAPMFIGKRASAPLFIGKRRAPMFVGKRVNKDWNELLSALHTLQAARHYR
RMIQADRRHNAPFLWAGGARLRKMGTSQTTRWKSKAITSGKMAGSRDARSAIVVVADLAKTVADVRTVHAIYVLDVDLTLLKVGLVFSVQRQTSYSQFLA*

>*Mytilus* inhitotory peptide (MIP)-2

MQGIGQLLVSTLLLLLPPLSVLAHKTGSDSFSKMQVDSEERVTSPALTSPSPTHRSSSSGGAADKAEAAAVVVASAPSSRTSQHPKLGPPLAVPEINQAFP
NSFSALTHHTLINDPFSTNLGPHTPYTAMEDTGPVQYQVNWNPESQSDVEFRVAEPEDEETTVDDEADNGKTTDIVENNDDYEPPKNLEDEDDSRITEQDG
ISPRQRVKRGGYTAPYFVGKRSVDENEGMDHDSQLQKRDTGYDQIPNGQDIPPQFTDKRRTPMFVGKRRTPMFVGKRRTPMFVGKRRTPMFVGKRRTPMFV
GKRRTPMFVGKRRTPMFVGKRRTPMFVGKRRTPMFVGKRRTPMFVGKRRTPMFVGKRRTPMFVGKRRTPMFVGKRRTPMFVGKRRTPMFVGKREDDAYPDD
AIELTDEENPVDTTQSASDTEKRAPTPMFVGKRSPTPMFVGKRSPTPMFVGKRSPAPMFVGRRGPTPMFVGRRGPTPMFVGRRGPTPMFVGKRSPTPMFVG
RRDPIPMFVGKRGESQTEDDPQFIEEADSAETNNDLQDEEQNSDDRDDQNSSESPDKRRTPMFVGKRRTPMFVGKRRTPMFVGKRRTPMFVGKRRTPMFVG
KRPTPMFVGKREKEGLSELLNALQTLQAARHYRRLIQTDKRHNAPFFIGRRSLPSTEDLTGQNTQSNFLR*

>NdWFamide-1

MVRSSAVLLVLVLASVVCTSHANWYGKRGNRGDFLSLLMQQRLDGLPSRDMTAEVALAAIDQILQLYRQHEAPQGAMDLKQGA*

>NdWFamide-2

MARISGAMLIVLVVLVSMAHISEANWYGKRSDRADFLNFLMKRDSVSRDMNPEVALAAIDQVLQLYRQRKADNSS*

>Neurokinin-Y1

MSSPAHHTLLTLFLSTLLFACVPARSLRDENLHTLRDIMAGNAEPDYYTFLHRLLSLVDEFVSKNSAADGMKRPWLDEEEVLLPPGFTKRVTWTPLGHLPA
SARIGRPQGGSRPHVEGPGTSVFRYG*

>Neurokinin-Y2

MTPCSHPLLSITATLLLLLLSSAGGRSLGGEPLAVSRPSGADMLSLLTRTSDPYDDPDLLLGLWNHLVSLRHVMPYDQPAVSLEDAKRSFPNGMKRKMVWS
PLGQLPASARLGRPQNQRPSSEDSSSPFFRYG*

>Neuroparsin

MSPSKVLSILVFTNLAAICLRCGNAATARSTNRQAFRKLFGCPSCEAADCPPLDPPGCASVLEPGVCGCCQVCARGQGESCGLASGRCGKGLACRPRPGDP
DPLGALLDGTAVCM*

>Neuropeptide (NP)-F1

MHKAFLSALLVISLVLAEVTCQEAMLAPPDRPQEFRSPGELRRYLKALNEYYAIVGRPRFGRSVSTNKRSVGDLGDLKADE*

>Neuropeptide (NP)-F2

MQKTLITLAVVVSLLLMGVTCQEELQPPIRPARFNSPEELKNYLRALNEYYAVMGRPRFGRSIPTQQKSSDAKSWTRD*

>Orcokinin-related peptide (partial)

-ISGTGVMGSFGGKRSFDPISRTGVMGSFGGKRSFDSISRTGEMGPVGKQSFDPMSDTGVQGPFEGKRSFN-

>Pedal peptide (Pedal-P)

MEKGLTAVVLVSLLAAAVWANDEKVVESSENDNMKKRSFDSINGNSRLNSFAKRPFDSISGNSGLSSFAKRPFDSISGNSGLSSFAKRPFDSINGNSRLSA
FAKRPFDSISGNSGLSSFAKRPFDSINGNSRLSAFAKRPFDSISGNSGLSSFAKRPFDSINGNSRLSAFAKRPFDSISGNSGLSSFAKRPFDSISGNSGLS
SFAKRPFDSISGNSGLSSFAKRPFDSISGNSGLSSFAKRPFDSISGNSGLSSFAKRPFDSISGNSRLSAFAKRSFDSINGNSRLNSFAKRPFDSISGNSGL
SSFAKRPFDSISGNSGLSSFAKRPSDSISGNSGLSSFAKRPFDSISGNSGLSSFAKRPFDSISGNSGLSSFAKRPFDSISGNSGLSSFAKRPFDSISGNSR
LSAFAKRPFDSISGNSGLSSFAKRPFDSISGNSGLSSFAKRSFDSKAGHSELNSFPEKDVDVEDSDEALSKEEDAENQVLDSIQHGGFSSLNKRSVDSAAH
SSLQKRSAEDGDQEAEVIENEE*

>PentaFVamide (P-FVa)

MECVKHKALFVLLSTLLVSLLLSPAFTEASARQQDGQKTPSTRDSSSGTDASADESLRDSSNSASEVENQSGFGQVVHERSSGDAHRKGETEKRLRDFVGK
RSSVEKEGDGVSHPARSVGGGEASSETGGQSAYFQSNNQEGSPLTKRSVDKRLRDFVGKRDGQSDNELDFTKRLRDFVGKRSVLYPSDIQKRLRNFVGKRE
VPEYSVDVQKRLRDFVGKRWGVAYPVGAYKRHRYFVGKRSVEPYSLDMQKRLRDFVGKREVLPYDVQKRLRDFVGKREAVGYRADVQKRLRDFVGKRSPMP
YYGEVQKRLRDFVGKRDYQNEVQKRLRDFVGKRDYQNEVQKRLRDFVGKRDYQNEVQKRLRDFVGKRDYQNDVQKRLRDFVGKRDYQNEVEKRLRDFVGKR
DSVQYDLEEVQKRLRDFVGKRGTPLFSSDIHKRIRDFVGKRALDSALAVDSPAEVMVYRDWEDDTVDDGSNLPSYMSDVPAPPEDSRDMEKRAPIREFVGK
RSGLYSDSSVPLSAENVDSEDFIPVDTPVEDDPSLTASKRLRDFVGKRADMDKRLRDFVGKRGSMNELLAGGSDDALPLQATNGGAGPEAFPLSKRLRDFV
GK*

>Pleurin

MKNMQAMVQTMAAVWVLFMVTGTTCAVFFTLSRDNNYPRIGKRVFFTRGDKGSSYPRIGRSSPGPEGHAAMTGSDGSFRSPPKRSAFGMRSAEELGGLDLS
APSVSASSADLMTSLEDPDVEGSEEQSWLPLGLLFYSFDKDGDKQLSRQEFIRGMINARESGPKCR*

Figure S4 (continued)

>Prohormone4-related peptide A (ProH4A)
MMTTVVLSFLLMMVHGCHFSTLLSQLQDQPVSSGHRKLSSHDGELLSRMQHLADDQPLPSGQTSHVILSAPDLITMPDVSLSWILFPGRRPQDTSIISKRA
ALLDFRQQSLSQELWLALMRYSRQSHDDIDNEDKVSKSVFPWDTRSSLWKMWPWYQQMLHLSSDNLQNALTEVEQHHKKNPNELPLKLQSSPYRQRTISAV
HNNNMFPALKSLLEDRDVHTGKNHVDVNIRSPHSPHHLDFTLQNSEEPDSGITTGLLNIFGSATSFSDQNYDKNLRFLHTADSGFNSMDKVWDLDLRDESA
SGAEEEEEEEEDDEAGSSSLPQSVLSSLDMKTFSGEEGEVDAAVGNREQDASEALRSTSWPQSGRLQQERDVVMVPLTILPLDMGKRGGGISREELVRRLH
RPHQLRSLVTLTHWSQRGRGRGREHEDKVRTRRRHRLLRLLEGIRLPRRRVRTVSRKGCTASGNTDAPVKRCPSDVDICLSDVMFCDGIRDCPKGEDEDAS
ICTLPQRNLQNSRSFTSFPNVRPLSSAIVRSSDS*

>Prohormone4-related peptide B (ProH4B)
MRSLTLPVFLLLLLLPTVHFSSLHPDLVYRHKLARAARLSDSDAMYQSVTAPRDLLHMSHRASASSDLHKKLRTNLVDNKDSNNLHHRPLPSSTSWPGMDE
DSVSDELPRRRARENFAKEPLDFSPSAATLRQQYPDSVRRSENIVLRKYNAVDSRRKPKRDQLSYMNPDMAVGSVKNSWLFHNSRRHPVARCNYTTLHRTR
RSLAPYPGYDTVGMNDHVVVSDKPSSSVDPVVLKHRLQKIYRLFLTDEPIEASDVLQNTLNEFTKRGDAEAISESQNALNSHVSAHDALSPTVYSHLAKEP
SSDGSLDHREAEIIAKEQPMDPYLIEYLETVRPTDRDDTRKEHRTTEASWLDVQRRRSEKDPAERGEEERVLSVDDVVELPAELRDDVGSEVNQPDLIMVH
RRRKLSRAEQVRRLYDPSHLGPLARFSLSLKGREGKGEGEGKAKKHPSSLHKQLAKAQPLKAISESASSSAASKIRECRQEDSSAMTCPSDVTVCLRDVMI
CDGVRDCNLGEDESPFLCMMRLWAFTLLNNVHSIFSAYVTQPSRGSTRHQSHDYRHDRSYRQSPSS*

>Sensorin-A1
MMKTTTPRCAALVLLLLLVHLSLVHCRSLGQLDHPEEGVNERLTEAGKRDGRRITRGRYRMGYMFGKRSELEGSTASLLVKNLIGKAMSVDELTRAMQSDA
TLAERVARHLDKNGDGYVAVGELL*

>Sensorin-A2
MQSASLCSLSLLLACLCVAAHCLPLDASEEQDTRRVARGRYKMGYMFGKRSELEGKAHTLLLGSLVGKAISVEDLGRVLQTDAALAHRMARHLDTNGDGYV
SVRELL*

>SGLVa
MAALRSPSLLLVCLAAWTVISLALTTSVTADAGADDRRKRQESLDELSSGLIGKRDHKTPHNKFFDKRPFGELASGLVGKRPFGELASGLVGKRPFGELAS
GLVGKRPFGELASGLVGKRPFGELASGLVGKRPFGELASGLVGKRPFGELASGLVGKRPFGELASGLVGKRPFGELASGLVGKRPFGELASGLVGKRPFGE
LASGLVGKRPFGELASGLVGKRPFGELASGLVGKRPFGELASGLVGKRPFGELASGLVGKRPFGELASGLVGKRPFGELASGLVGKRPFGELASGLVGKRP
FGELASGLVGKRPFDNLASGLVGKRPFGELASGLVGKREFGDPASGLVGKRPFGELASGLVGKRPFGELASGLVGKRPFGELASGLVGKRPFGELASGLVG
KRPFGELASGLVGKRPFGELASGLVGKRPFGELASGLVGKRPFGELASGLVGKRPFGELASGLVGKRETSSS*

>Small cardioactive peptide (SCP)-B1
MDTTTFRIVFALVFVTVTVSEAASEVQEPRAKRSSYLAFPRLGRGYLALPRMGRSQPGSDAAVANGGSCCTTGLKKEWLFGDDGKMTAQNVCEAESCCAEF
LEVENTKPDGTFYSMCIPKCSLGDRVSAGK*

>Small cardioactive peptide (SCP)-B2
MDTTTFRIVFALVFVTVTVSEAASYLAFPRLGRGHLALPRMGRSQPGSDAAVANGGSCCTTGLKKEWLFGDDGKMTAQNVCEAESCCAEFLEVENTKPDGT
FYSMCIPKCSLGDRSLDAARDNMMLKLKRLLRN*

>SPamide
MHLLISALCAVAVLHLSIATVSADKASDSAGFRQIGSKIRHKRNSDSKVDLDALKDSLASVEDDLSNFQKRSCAVGLNSHHCAIAHMDDFMMGKDYLSGGF
SPGKRSTELPEDLRSQAEILLQNMSEQQQAVRALRSLLNNLGVRSKQQQKRTCYFRLGNHCLTEALDRAANQYYYLQSYQSPGRRRRSADQGSKVQSSPLS
SSSFSSSHGVERR*

>SXFY-1 (partial)
MRRIGGMYSGMVWGAMSPVCSAMTGLLMLLMLLLTCLSCAATASSELTVCSKASSVCSLVTEAHSPRCACPEDQVCQASFRVTDTLWYGTCSELPECGVNQ
APVVLSADKTSVAEVLCRCPRIAESLQVWTDEATFLKHPYCQDSSNEADDSQLVDPHPEDQNAEQLSESSSEEQDPPSLSIKNLAHAEEDLADDSQELKGL
DYDSSASSPADDDLGKHRVDEASAFYKRGREDFNAFYKRARDSISAFYKRTPAGKKRFYKRSPRDVSAFYKRAPSDVSGFYKRGPNDVSGFYKRGPSDVSA
FYKRGRDDVSRFYKRDVSAFYKRGLDDVSGFYKRAPDGLRAFYKRAPSDVSGFYKRAPNDLSGFYKRSSDDVSSFYKRAPEEHSSFYKRALDDLSAFYKRG
LNDQSTFYKRGLNDQSAFYKRGPRVNDQSTFYKRGLNDQSAFYKRGPSDLSAFYKRGPSDLSDLSAFYKRGLNDQ-

>SXFY-2 (partial)
-DQSSFYKWAPGDLSAFYKRARDENSGFYKRGVDDVSGFYKRARNDNSRFYKRDPNDVSAFYKRARNDNDSFYKRALDEVSSFYKRARDDTSSFYKRARDD
TSSFYKRARDDTSSFYKRARDDTSSFYKRARDDTSSFYKRSPNDLSAFYKRSGDDDLRSFYKRSADDHSRFYKRAGLSDSAFYKRQSGEDQTSDVASAS*

>Tachykinin-1
MTPRHMTSLVAIFVCNLLLSCAQSTPNAALLKLLAAKHSHDSSVSLPPSSSFSSYISHNYNAAFPAASRAADDVNALWLDSAGSSPVLMRGGNSLTSAGSS
TTPEEEAEEAKEGLLAASKRFHPSAFFGSRGKREYEGLTDAYAKRFFHQGFVGSRGKRLPAYLLNGGRMGEGEEGEQDEEEGEEEEEEEEQGLAGKRMISK
KFDPSGFTGSRGKRYLPGLEALFLSQMYKRLDAEKWKRQSLLGFHAARG*

>Tachykinin-2
MRSLTSSWSLCGLAGHKVTAVAAVVMCHFLLTSARSVPEDNGPLTMLTPGGAETEVDDKAVESANHRAIPKVMVEAQKRLEERGMTPDLYKRFIQGFVGSR
GKRAALMDDLMHGRGMSEDALSKRFIQGFVGSRGKRVLEGGDSLPMAASVFKRFMPGGYHASRGKRYMPAGLQALWHSQLHQKHEY*

Figure S4 (continued)

>Tetradecapeptide (TDP)-1

MMRTSLVLLALIVLALGDALPTKNLSRQKRGFRVNAAGRVAHGYGKRQFNTWEDNPLKGQPASQLMTVSELAQLAVDNPSLTEALIEKFIDLDGDGIVSSQ
ELFGLDAQ*

>Tetradecapeptide (TDP)-2

MRASLVFLALVVIALCQALPTQSHSRQKRGFRMNSSNRVAHGFGKRQSEAVDDNLFAILPSSDLMTVQELAQLSVENPSLTEALLQKFVDTDGDGIVTTQE
LFGIAAD*

>*Thais* Enterin-related peptide (TERP)-1

MWTLRALACGTQLLLILLLSFCCCLATAEAESSSADAQPRSLTKRSLADDLLDSAEDADLYNEPDFDLDDLMEKRTPYGYSARAPTFGHLFVGKRQPAFS
YRFVGKRLPKGITVRVPSFGHRFVGKRIPDNLDIRAPTFGHRFVGKREDEEGLDDLDLPLFPHSIRKRFAEDDFEDLAIDTRAPTFGHRFVGKRLRDYRRR
APSFGHRFVGKRQPSFGHRFVGKRIPDGLVERVPSFGHRFVGKRESEDLEKRLPTFGHHFVGKRVPDDADKRVPSFGHRFVGKRAADDSETMLPTSFAGDM
NAPVVSHSIRKRFAEDDRAEALAVDTRAPSFGHRFVGKRMLPGFEKRAPSFGHRFVGKRQPSFDHSFVGKRIPDGLAERVPSFGHRFVGKRVADDLDKRVP
TFGHHFVGKREDDKAEKRKPSFGHMFVGKRESGDLDNELPVFEHNLAATMMADDLDKRIPSFGHRFVGKRDSSDQTKRVPAFGHRFVGKRIPDGLEVRAPS
FGHRFVGKRIPEEADKRVPSFGHMFVGKRLSPSIDTRVPQFGHRFVGKRSEDESAQRDSSGSGDKITSPSPMTSQPLKQ*

>*Thais* Enterin-related peptide (TERP)-2

MMACRDLFSSRQFLLLLLFFLLSALLSTSLASAEEDDSDSATDLEKRSLPLFGNHYLYKRLLRLPFQKRQFPSRFQHSFIGKRSEDDDLEDDFSDQLEEPA
DLEDPEVDKRRSFFTHRFVGKRIPDGLDIRGPGFIHRFVGKRPAGFMHRFVGKRVPDGLEERAGFSHRFIGKRVPLGLEYRARNFHHRFVGKRTPDGFDLR
APGFSHRFIGKRFMSGFNLRGPSFVHRFVGKRTPDGIDQRAPGFTHRFIGKRIPDGLDIRGPSFIHRFVGKRAPDGFDLKAGFSHRFVGKREDDDSEDFDD
SDYPHSIRKRFAENEGGENIHLDSRGSGFMHRFVGKRVPDGLEERAARYSHRYFGKRAPYGLEARAPYTHRFIGKRGFVHKFVGKRLSEDPEKRGPGFSHR
FVGKRISEELDKRASFSHRFVGKRDPDEVDKRASFSHRFVGKRVPDGIDERSPGFSHRFVGKRVPDGTDERAPGVTYKFMGKRLDDGIDERGFSHRFVGKR
SEESPQSEGSSQTQADAASSKRATS*

>*Thais* excitatory peptide (TEP)-1

MTTRGSCVMCAAMMSLLILSSIVSNAEGKCSGKWAIHACWGGNGKRSGIPAEEDDNTAKSLDLLRRLLRRKVSSSPYLPSLEDTVYVPVQPEDFLSSSESL
PSSLSSSSSLLSSSSPFEDGFSEDVEEAEAAAASFVPSKNRLASLTTILRKLQQGRDDFQ*

>*Thais* excitatory peptide (TEP)-2

MKTDIQQALCLAMTLFVIVSTVIKPSEGKCYGKWAMHACWGGNGKRSDPSADLAPNPSVLRQLLLRNPPALDSREDASSYSDGLPEYNVPPPPAPSVSRLA
ALLRTLRTLQRENDALP*

>VHGY

MKRHLVLLLVLTSVLTTSLTRAAPSQKTDAAAHSKVRDARDVSSSGDVVRLLTHLSSKQDDDSKAGDVSKRYLDTLGGFQVHGYKRSHEDNQEDGQEDSKD
SSSKRGFDDLGGFNVHGYKKRGFDDLGGFNVHGYKRGFDDLGGFNVHGYKKRGFDDLGGFNVHGYKRGFDDLGGFNVHGYKKRGFDDLGGFNVHGYKRGFD
DLGGFNVHGYKRGFDDLGGFNVHGYKRGFDDLGGFNVHGYKRGFDDLGGFNVHGYKRGFDDLGGFNVHGYKRGFDDLGGFNVHGYKRGFDDLGGFNVHGYK
KRGFDDLGGFNVHGYKRSEDKEDGSEEKDLKRGLDDLGGFRVHGLKKRAPETSTA*

>Whitnin

MEQQMMVMSAMLMVLLVSCACALPADDSMLQRSALSVDKRPKYMDTRDLDIFKYMLMASIRDLVEDGQLNSGVLSQGGEDDQSELKAVAKRMQYMGICMRR
RQNTFIPYPCLRTA*

>WWamide (B-type allatostatin)

MEIRYYSCLLLSALATLYVQLVEGMVEASLDQADSSPRDSQDLLRDVLNKRWPQFRSWGKKWDWKRWQALNTWGKRWNNQFATWGKRGWNQRPFATWGKRG
WDNRNFATWGKRGWDDVSFAPWGKRGWDDRSFATWGKRGWDDRSFAPWGKRGWDDRSFATWGKRGWDDRSFAPWGKRGWDDRSFAPWGKRGWDDRSFATWG
KRGWDDRSFTSWGKREDDEQHIGEADKRTKWRPAHYSMWIKRPTEWSKLGIWGKRGWGNNQFSTWGKRDGKEDTDILSNEEALSNGDAVDKREWNQFLTWG
KRQDLHHISKRRAEEQSRQTAIDTKDKQPAQSDNPEEPTKDISDANDNISEDNAESSEGSGSSNSNSSSKTDNAEDKHTDTDHKHGTDNVQRETAENKDKR
WKTMATWGKRQQPLSAASKKWSDMKVWGKRSVQDPELSKKWSHVSTWGKRWGNVAVGGKQDDSMDKKWKSMSVWGKRDEPMDKKWKSMSVWGKRDQPMDKK
WKSMSVWGKRDDSMNKKWKSMSVWGKRDDSMDKKWKSMSVWGKRDQPMDKKWKSMSVWGKRDQPMDKKWKSMSVWGKRDQPTDKKWKSMSVWGKRDQPMDK
KWKSMSVWGKRDEPMDKKWKNMAVWGKRLFPKRWASVSTWGKRSHDTTDTQEADKKWSGLSTWGKRDPAEENSMDKKWSNMAVWGKRQADQQSPLETDKRW
NALTSWGKRSGHGDLPSAEGKEGAESNPEADKQAAKDFLFQFADTDGNRQVDDQELQHFMGWLKEEQQSSQL*

Figure S4 (continued)

1.2 Supplementary Tables

Supplemental Table 1. Classification of annotated clusters up-regulated in OK snail.

Category	#	Sub-category 1	#	Sub-category 2	#
Enzyme	53	Reductase	7		
		Synthase	7		
		Protease	6		
		Kinase	5		
		Isomerase	4		
		Hydrolase	4		
		Transferase	4		
		Phosphatase	3		
		Protease inhibitor	3		
		Sulfatase	2		
		Dehydrogenase	2		
		Cytochorome-P450	2		
		others	4		
Signaling	26	Signal transduction	12	Small GTPase	8
				Tyr-kinase	3
				JNK	1
		Receptor	9	Immune response	5
				GPCR	2
				Ionotropic	1
				Adhesion	1
		Ligand	2	Pheromone	1
				Peptide	1
Transporter	16	Amino acid	3		
		Bicarbonate	3		
		Ion	3		
		Monocarboxylate	2		
		Lipid	2		
		others	3		
ECM	18	Collagen	9		
		Membrane protein	7		
		others	2		
Gene expression	10	DNA replication	3		
		Nuclear-receptor associated	3		
		RNA synthesis	2		
		others	2		
Ubiquitination	9				
Chaperone	8				
Vesicle trafficking	7				
Cytoskeleton	6				
Stress response	5				
Apoptosis	3				
Cell division	3				
others	8				

Supplemental Table 2. Classification of annotated clusters down-regulated in OK snail.

Category	#	Sub-category 1	#	Sub-category 2	#
Signaling	51	Signal transduction	9	Small GTPase	1
				Tyr-kinase	2
				Others	6
		Receptor	9	GPCR	8
				Ionotropic	1
		Ligand	33	Peptide	33
Enzyme	43	Protease	19		
		Transferase	7		
		Esterase	6		
		Sulfatase	3		
		Dehydrogenase	2		
		Oxidase	2		
		others	4		
Transporter	13	Amino acid	4		
		Ion	3		
		others	6		
Gene expression	13	RNA-processing	8		
		Transposon	3		
		others	2		
Transmembrane	7	Schaffold	4		
		others	3		
Channel	5	Hydrogen	3		
		Potassium	2		
ECM	5	Adhesion	3		
		Gollagen	1		
		others	1		
Vesicle trafficking	4				
Ubiquitination	4				
Cytoskeleton	1				
Apoptosis	1				
uncharacterized	3				
others	10				

Supplemental Table 3. Neuropeptide precursors of *Reishia clavigera* identified by homology search.

Peptide name	Abbreviation	Cluster encoding neuropeptide precursor	Hit by BLAST on database				Hit by local BLAST on clusters		
			Entry name	Accession #	Score	e-value	Query	Score	e-value
Achatin-1		>Cluster-112285.180918_TRINITY_DN7257_c2_g1_i2					AQS80481.1 achatin-1 precursor [Charonia tritonis]	107.0	1.00.E-28
Achatina cardioexcitatory peptide	ACEP	>Cluster-112285.199728_TRINITY_DN86612_c0_g1_i16					BAA76406.1 ACEP-1 precursor [Lissachatina fulica]	42.0	1.00.E-04
Adipokinetic hormone	AKH	>Cluster-112285.52383_TRINITY_DN3339_c0_g1_i1					AQS80483.1 adipokinetic hormone, partial [Charonia tritonis]	32.7	4.70.E-02
Allatostatin-A1		>Cluster-112285.96403_TRINITY_DN15874_c0_g1_i1	BUCC_APLCA	P20481	50.4	1.79E-82			
Allatostatin-A2		>Cluster-112285.67224_TRINITY_DN1827_c1_g1_i29	BUCC_APLCA	P20481	186.0	3.88E-51			
APGWamide-1	APGWa-1	>Cluster-112285.100173_TRINITY_DN8282_c0_g1_i1					AQS80486.1 APGWamide precursor [Charonia tritonis]	145.0	9.00.E-41
APGWamide-2	APGWa-2	>Cluster-112285.91209_TRINITY_DN5376_c1_g1_i24	CP1_APLCA	Q10998	147.0	1.14E-43			
Buccalin		>Cluster-112285.95650_TRINITY_DN3777_c1_g1_i16	BUCC_APLCA	P20481	97.4	8.35E-23			
Bursicon							AQS80492.1 bursicon-beta precursor [Charonia tritonis]	No hit	
Cerebrin		>Cluster-112285.112273_TRINITY_DN712_c0_g1_i4					AQS80497.1 Cerebrin precursor [Charonia tritonis]	129.0	1.00.E-35
Cholecystokinin-1	CCK-1	Cluster-112285.81104_TRINITY_DN279_c3_g1_i3					AQS80495.1 CCK-1 precursor	110.0	5.00.E-29
Cholecystokinin-2	CCK-2	Cluster-112285.77969_TRINITY_DN10408_c5_g1_i6					AQS80496.1 CCK-2 precursor	101.0	1.00.E-25
Clionin-1		Cluster-112285.88880_TRINITY_DN676_c0_g1_i13					ABU82762.1, Clionin [Tritonia tetraquetra]	57.8	6.00.E-10
Clionin-2		Cluster-112285.125487_TRINITY_DN6985_c0_g1_i4					ABU82762.1, Clionin [Tritonia tetraquetra]	53.5	1.00.E-08
Conopressin		Cluster-112285.186720_TRINITY_DN4598_c0_g1_i1					AQS80498.1 Conopressin precursor [Charonia tritonis]	139.0	3.00.E-39
Crustacean cardioactive peptide	CCAP	Cluster-112285.96308_TRINITY_DN5975_c0_g1_i16	CCAP_CONVL	E3PQQ8	193.0	7.66E-61			
Egg-laying hormone	ELH	Cluster-112285.95686_TRINITY_DN7518_c0_g1_i10					AQS80499.1 ELH precursor [Charonia tritonis]	74.7	2.00.E-15
Elevenin-1L		Cluster-112285.114937_TRINITY_DN5053_c2_g1_i5					AQS80540.1 Elevenin [Charonia tritonis]	66.6	3.00.E-13
Elevenin-1S		Cluster-112285.141404_TRINITY_DN67194_c0_g1_i5					AQS80540.1 Elevenin [Charonia tritonis]	30.4	1.40.E+00
Elevenin-2		Cluster-112285.92165_TRINITY_DN10930_c0_g1_i2					AQS80540.1 Elevenin [Charonia tritonis]	69.3	5.00.E-14
FFamide-1	FFa-1	Cluster-112285.87434_TRINITY_DN4557_c0_g2_i1					AQS80504.1 FFa [Charonia tritonis]	94.7	2.00.E-23
FFamide-2	FFa-2	Cluster-112285.136782_TRINITY_DN41896_c0_g1_i1					AQS80504.1 FFa [Charonia tritonis]	90.9	7.00.E-23
FLRFamide/FMRFamide	FLRFa/FMRFa	Cluster-112285.200507_TRINITY_DN3517_c0_g2_i4					AQS80505.1 FMRFamide precursor, partial [Charonia tritonis]	122.0	7.00.E-33
FMRG		Cluster-112285.133659_TRINITY_DN6365_c0_g1_i4	FMRF_APLCA	P08021	52.8	4.15E-06			
FRFamide	FRFa	Cluster-112285.232181_TRINITY_DN6092_c0_g1_i2					AQS80524.1 LFRFamide-1 precursor [Charonia tritonis]	191.0	3.00.E-59
FRYamide	FRYa	Cluster-112285.91197_TRINITY_DN1329_c0_g1_i11					AQS80526.1 LFRYamide precursor [Charonia tritonis]	66.6	7.00.E-13
Fulicin		Cluster-112285.87930_TRINITY_DN2862_c0_g2_i3	FULI_LISFU	P35905	64.7	8.07E-11			
FVRIamide-1	FVRIa-1	Cluster-112285.121291_TRINITY_DN2917_c0_g1_i9					AQS80508.1 FxRIamide-1 precursor [Charonia tritonis]	179.0	8.00.E-51
FVRIamide-2	FVRIa-2	Cluster-112285.127875_TRINITY_DN5484_c1_g1_i5					AQS80509.1 FxRIamide-2 precursor [Charonia tritonis]	253.0	3.00.E-81
FXXFamide	FXXFa	Cluster-112285.204001_TRINITY_DN1007_c0_g1_i8					AQS80506.1 FMRFamide neuropeptide-like precursor [Charonia tritonis]	270.0	1.00.E-79
GDPFLRFamide	GDPFLRFa	Cluster-112285.142183_TRINITY_DN8884_c8_g2_i2					sp|P42565.1 GDPFLRFa Precursor [Lymnaea stagnais]	59.7	6.00.E-09
GGKVamide	GGKVa	Cluster-115334.1_TRINITY_DN17990_c0_g1_i1	DDRGK_MOUSE	Q80WW9	33.5	1.50.E+00			
Glycoprotein hormone-α	GpH-α	Cluster-112285.105225_TRINITY_DN5684_c0_g1_i9					AQS80514.1 GPA2 precursor [Charonia tritonis]	160.0	1.00.E-47
Glycoprotein hormone-β	GpH-β	Cluster-112285.59760_TRINITY_DN9104_c5_g1_i1	CTHB5_CONVC	A0A0F7YZI5	214.0	1.63E-70			
Gonadotropin-releasing hormone	GnRH	Cluster-112285.212703_TRINITY_DN41231_c0_g1_i6	GRHLP_RUDPH	A0A0A7DNP6	45.8	3.78E-06			
Gonadotropin-releasing hormone-RP	GnRH-RP	Cluster-112285.111845_TRINITY_DN1711					AQS80512.1 GnRH variant-A precursor [Charonia tritonis]	36.2	2.40.E-02
Helicostatin-RP		Cluster-112285.188186_TRINITY_DN3951_c0_g1_i61					AQS80535.1 PXFVamide-1 precursor, partial [Charonia tritonis]	70.5	8.00.E-12
Helix command specific 2	HCS2	Cluster-112285.129474_TRINITY_DN3624_c0_g1_i28	CNP_HELLU	Q25077	85.9	3.11E-20			
Insulin-like 1A	Insulin-1A	Cluster-112285.101436_TRINITY_DN6004_c0_g1_i1					AQS80516.1 insulin-like 1A precursor [Charonia tritonis]	181.0	2.00.E-52
Insulin-like 1B	Insulin-1B	Cluster-112285.128304_TRINITY_DN1105_c10_g1_i2					AQS80517.1 insulin-like 1B precursor [Charonia tritonis]	48.5	2.00.E-06
insulin-like 2	Insulin-2	Cluster-112285.94663_TRINITY_DN1105_c10_g1_i5					AQS80518.1 insulin-like 2A precursor [Charonia tritonis]	115.0	9.00.E-31
Leucokinin		Cluster-112285.83113_TRINITY_DN2736_c0_g1_i25					ATD50219.1 leucokinin [Aplysia californica]	188.0	1.00.E-46
Maturation associated peptide-1A	MAP-1A	Cluster-112285.12049_TRINITY_DN6494_c0_g1_i21					TBIU010983 MAP-1 [Haliotis discus hannai]	170.0	1.00.E-48
Maturation associated peptide-1B	MAP-1B	Cluster-112285.188387_TRINITY_DN11793_c0_g1_i3					TBIU010983 MAP-1 [Haliotis discus hannai]	154.0	9.00.E-43
Maturation associated peptide-2A	MAP-2A	Cluster-112285.247400_TRINITY_DN8136_c1_g1_i2					TBIU005609 MAP-2 [Haliotis discus hannai]	50.4	4.00.E-07
Maturation associated peptide-2B	MAP-2B	Cluster-112285.125306_TRINITY_DN5923_c0_g1_i6					TBIU005609 MAP-2 [Haliotis discus hannai]	44.7	4.00.E-05
Maturation associated peptide-2C	MAP-2C	Cluster-112285.65329_TRINITY_DN23757_c0_g1_i6					TBIU005609 MAP-2 [Haliotis discus hannai]	40.8	1.00.E-03
Maturation associated peptide-3	MAP-3	Cluster-112285.116199_TRINITY_DN3268_c0_g1_i11					TBIU009796 MAP-3 [Haliotis discus hannai]	112.0	5.00.E-29
Maturation associated peptide-4	MAP-4						TBIU003144 MAP-4 [Haliotis discus hannai]	No hit	
Maturation associated peptide-5A	MAP-5A	Cluster-112285.112080_TRINITY_DN3457_c0_g1_i11					TBIU013612 MAP-5 [Haliotis discus hannai]	94.4	1.00.E-22
Maturation associated peptide-5B	MAP-5B	Cluster-112285.173333_TRINITY_DN230_c1_g1_i1					TBIU013612 MAP-5 [Haliotis discus hannai]	93.6	4.00.E-22
Maturation associated peptide-6A	MAP-6A	Cluster-112285.165091_TRINITY_DN3729_c0_g1_i1					TBIU017101 MAP-6 [Haliotis discus hannai]	62.8	1.00.E-11
Maturation associated peptide-6B	MAP-6B	Cluster-112285.85243_TRINITY_DN50797_c0_g1_i1					TBIU017101 MAP-6 [Haliotis discus hannai]	60.1	8.00.E-11
Maturation associated peptide-7	MAP-7						TBIU032784 MAP-7 [Haliotis discus hannai]	No hit	
Maturation associated peptide-8	MAP-8	Cluster-112285.56264_TRINITY_DN52693_c0_g1_i2					TBIU035555 MAP-8 [Haliotis discus hannai]	36.6	8.00.E-03
Microseminoprotein	MSP	Cluster-112285.34421_TRINITY_DN2282_c2_g2_i2	MSMB_DORPE	D2X5V5	50.1	5.14E-08			
Myomodulin		Cluster-112285.74499_TRINITY_DN15549_c0_g1_i1					AQS80527.1 myomodulin-1 precursor [Charonia tritonis]	206.0	2.00.E-61
Mytilus inhibitory peptide-1	MIP-1	Cluster-112285.91686_TRINITY_DN11_c5_g1_i2					AQS80536.1 PXFVamide-2 precursor, partial [Charonia tritonis]	186.0	9.00.E-53
Mytilus inhibitory peptide-2	MIP-2	Cluster-112285.87449_TRINITY_DN2729_c3_g1_i1	MIPR_APLCA	Q9NDE8	68.6	3.25E-11			
NdWFamide-1	NdWFa-1	Cluster-112285.127264_TRINITY_DN10996_c1_g1_i1					AQS80529.1 NdWFamide-1 precursor [Charonia tritonis]	112.0	9.00.E-31
NdWFamide-2	NdWFa-2	Cluster-112285.94925_TRINITY_DN4475_c17_g1_i1					AQS80530.1 NdWFamide-2 precursor, partial [Charonia tritonis]	62.8	4.00.E-13
Neurokinin-Y1		Cluster-112285.180708_TRINITY_DN19971_c1_g1_i3					AQS80531.1 NKY [Charonia tritonis]	100.0	7.00.E-26
Neurokinin-Y2		Cluster-112285.126615_TRINITY_DN5772_c2_g1_i1					AQS80531.1 NKY [Charonia tritonis]	59.3	2.00.E-10
Neuroparsin		Cluster-112285.222261_TRINITY_DN1621_c0_g1_i1					NP_001124362.1 neuroparsin precursor [Bombyx mori]	37.7	2.00.E-03
Neuropeptide-F1	NP-F1	Cluster-112285.114938_TRINITY_DN13873_c1_g1_i5					EB290938.1 NPF_A.californica_EST	66.2	1.00.E-13
Neuropeptide-F2	NP-F2	Cluster-112285.90019_TRINITY_DN2297_c0_g1_i11					NP_001191635 NPY_A.californica	61.6	9.00.E-12
Orcokinin-RP		Cluster-112285.93519_TRINITY_DN3911_c1_g1_i24	YV023_HUMAN	A8MUU9	63.2	1.18E-10			
Pedal peptide	Pedal-P	Cluster-112285.108505_TRINITY_DN5712_c0_g1_i3					NP_001191585.1 pedal peptide-1 precursor [Aplysia californica]	277.0	8.00.E-84
PentaFVamide	PeFVa	Cluster-112285.95473_TRINITY_DN4682_c1_g1_i5	PRQFV_APLCA	Q86MA7	176.0	8.25E-47			
Pleurin		Cluster-112285.192806_TRINITY_DN35450_c0_g1_i3					AQS80533.1 Pleurin [Charonia tritonis]	103.0	4.00E-26
Prohormone4-A	ProH4A	Cluster-97277.0_TRINITY_DN23117_c0_g1_i23	PROH4_APIME	P85831	39.7	7.00.E-03			
Prohormone4-B	ProH4B	Cluster-112285.165481_TRINITY_DN162292_c0_g1_i8	PROH4_APIME	P85831	41.6	7.00.E-03			
Sensorin-A1		Cluster-112285.95415_TRINITY_DN7017_c2_g1_i6					CAA40089.1 Sensorin A [Aplysia californica]	63.5	4.00.E-12
Sensorin-A2		Cluster-112285.116599_TRINITY_DN3307_c0_g1_i1					CAA40089.1 Sensorin A [Aplysia californica]	58.2	3.00.E-10
SGLVamide	SGLVa	Cluster-112285.155580_TRINITY_DN50432_c0_g1_i27					AQS80523.1 LASGLVamide-4 precursor, partial [Charonia tritonis]	77.0	6.00.E-17
Small cardioactive peptide-1	SCP-1	Cluster-112285.129558_TRINITY_DN356_c2_g1_i3					AQS80537.1 sCAP-1 precursor [Charonia tritonis]	154.0	1.00.E-43
Small cardioactive peptide-2	SCP-2	Cluster-112285.129555_TRINITY_DN5353_c0_g1_i26					AQS80538.1 sCAP-2 precursor [Charonia tritonis]	142.0	2.00.E-39
SPamide	SPa	Cluster-112285.124728_TRINITY_DN785_c3_g1_i5					XP_046579299.1 uncharacterized protein LOC124286904 [Haliotis rubra	142.0	1.00.E-39
SXFY-1		Cluster-112285.110536_TRINITY_DN17540_c1_g1_i15					AQS80502.1 FCAP-1 precursor, partial [Charonia tritonis]	164.0	3.00.E-44
SXFY-2		Cluster-112285.119666_TRINITY_DN3892_c3_g1_i18					AQS80502.1 FCAP-1 precursor, partial [Charonia tritonis]	293.0	4.00.E-91
Tachykinin-1		Cluster-112285.188624_TRINITY_DN12191_c0_g1_i5					ARS01403.1 tachykinin 1 [Deroceras reticulatum]	33.9	1.40.E-01
Tachykinin-2		Cluster-112285.130766_TRINITY_DN10428_c0_g1_i7					ARS01404.1 tachykinin 2 [Deroceras reticulatum]	39.3	3.00.E-03
Tetradecapeptide-1	TDP-1	Cluster-112285.92164_TRINITY_DN2890_c0_g2_i1					ARS01354.1 allatotropin 1 [Deroceras reticulatum]	46.6	6.00.E-06
Tetradecapeptide-2	TDP-2	Cluster-112285.142348_TRINITY_DN3754_c0_g1_i3					ARS01355.1 allatotropin 2 [Deroceras reticulatum]	81.6	3.00.E-18
Thais enterin related peptide-1	TERP-1	Cluster-112285.91962_TRINITY_DN5166_c0_g1_i3					AQS80500.1 Enterin-1 precursor [Charonia tritonis]	433.0	4.00.E-139
Thais enterin related peptide-2	TERP-2	Cluster-112285.85242_TRINITY_DN1518_c0_g1_i10					AQS80501.1 Enterin-2 precursor [Charonia tritonis]	589.0	0.00.E+00
Thais excitatory peptide-1	TEP-1	Cluster-112285.91990_TRINITY_DN1169_c2_g1_i2					BAQ25802.1 TEP-1 precursor [Reishia clavigera]	160.0	5.00.E-49
Thais excitatory peptide-2	TEP-2	Cluster-112285.89680_TRINITY_DN49862_c3_g1_i1					BAQ25803.1 TEP-2 precursor [Reishia clavigera]	202.0	5.00.E-63
VHGY		Cluster-112285.146472_TRINITY_DN164438_c0_g1_i9	FCAP_APLCA	Q8ISH7	175.0	9.13E-50			
Whitnin		Cluster-112285.93268_TRINITY_DN14743_c0_g1_i2					AQS80532.1 PKYMDT precursor, partial [Charonia tritonis]	119.0	4.00E-33
WWamide	WWa	Cluster-112285.93035_TRINITY_DN3424_c11_g2_i2					AQS80539.1 WWamide precursor, partial [Charonia tritonis]	149.0	2.00.E-38

RP : related peptide

Supplemental Table 4. Mature neuropeptides identified by the nanoLC-Orbitrap MS/MS analysis on *Reishia* ganglia.

Name	Structure	a.a	Precise molelular mass					RT	RMSE
			caliculated	PTM	measured	detected (m/Z)	Z	(min)	(ppm)
ACEP	APSWRPQGRFa	10	1199.6313	Am	1199.6312	400.8840	3	39.28	-0.8
AKH	pQIHFSHSWGTa	10	1180.5380	Am, Py	1180.5414	591.2819	2	54.98	6.5
Allatostatin-A1	GVDRNNFGFGLa	11	1193.5942	Am	1193.5941	597.8092	2	58.93	8.2
	GVDHNNFGFGLa	11	1174.5520	Am	1174.5519	588.2872	2	58.68	6.8
	NVDRNNFGFGLa	11	1250.6137	Am	1250.6156	626.3189	2	58.58	6.1
Allatostatin-A2	RLDRINFHVGLa	11	1337.7681	Am	1337.7679	446.9316	3	51.73	3.9
	TIDRTNFRFGLa	12	1337.7205	Am	1337.7205	669.8678	2	52.19	0.4
	pQIDRNSFRFGLa	11	1350.7157	Am, Py	1333.6891	667.8513	2	63.38	-0.8
	TIDQAGFRFGLa	11	1222.6459	Am	1222.6459	612.3347	2	62.76	7.4
	KVDHNNFGFGLa	11	1245.6255	Am	1245.6254	623.8201	2	48.57	0.2
	GVDHNNFGFGLa	11	1174.5520	Am	1174.5519	588.2872	2	58.68	6.8
	GVDRNNFGFGLa	11	1193.5942	Am	1193.5941	597.8092	2	89.89	8.2
	NVDRNNFGFGLa	11	1250.6157	Am	1250.6156	626.3189	2	58.58	6.1
APGWa-1	AEDLSSLDGLLDGEQFELVa	19	2047.9950	Am	2047.9949	1025.0140	2	90.27	9.1
Buccalin	GEKPGMDRLAFFGRLa	15	1691.8930	Am	1691.8929	564.9761	3	60.59	8.0
	GMDEYAFMGRLa	11	1287.5741	Am	1287.5740	644.7947	2	65.04	0.6
	PMDNRMFGPQLa	11	1303.6166	Am	1303.6166	652.8212	2	55.94	8.7
	pERIDNMMFGPQLa	12	1430.6805	Am, Py	1430.6798	716.3536	2	74.01	8.9
	ERMDNMMFGPQLa	12	1466.6469	Am	1466.6469	734.3354	2	63.43	6.5
	pERMDNMMFGPQLa	12	1448.6369	Am, Py	1448.6364	725.3318	2	72.52	8.8
	RMDNMMFGPQLa	11	1337.6043	Am	1337.6042	669.8087	2	59.16	-1.0
	ERIDGRMFGPQLa	12	1416.7297	Am	1416.7296	709.3784	2	52.18	8.9
	pERIDGRMFGPQLa	12	1398.7197	Am, Py	1398.7190	700.3706	2	59.76	5.5
	ERMDSMMFGPQLa	12	1439.6360	Am	1439.6360	720.8306	2	64.13	7.4
	pERMDSMMFGPQLa	12	1421.6260	Am, Py	1421.6254	711.8261	2	73.32	8.7
	pERMDSRMFGPQLa	12	1446.6866	Am, Py	1446.6860	724.3550	2	58.49	6.5
	ERMDQFMFGSRLa	12	1514.7123	Am	1514.7122	758.3696	2	61.29	8.3
	pERMDQFMFGSRLa	12	1496.7023	Am, Py	1496.7017	749.3649	2	68.27	9.1
CCAP	SLFSPWVNKMNGVVGEa	16	1761.8873	Am	1761.8872	881.9586	2	75.27	8.7
Cerebrin	NGGTLDTLYNLPNLNDIa	17	1844.9268	Am	1844.9269	923.4713	2	79.08	0.6
FLRFamide /FMRFamide	FLRFa	4	580.3486	Am	580.3485	291.1819	2	44.02	1.1
	ALTDDHFLRFa	10	1232.6303	Am	1232.6301	617.3262	2	58.48	6.2
	FMRFa	4	598.3050	Am	598.3050	300.1597	2	49.28	-0.3
FMRG	FMRGVDAYIRAa	11	1296.6762	Am	1296.6760	649.3503	2	50.91	7.7
	FMRGVDAYIKAa	11	1268.6700	Am	1268.6699	423.8998	3	49.60	6.1
	FMRGVDAYIQAA	11	1340.6548	Am	1340.6547	671.2401	2	59.14	8.2
FRFamide	GSLFRFa	6	724.4020	Am	724.4020	363.2081	2	48.93	-0.5
	SSLFRFa	6	754.4127	Am	754.4126	378.2158	2	55.08	5.8
	DVKSFHWa	7	916.4556	Am	916.4555	306.4922	3	96.00	-0.8
FRYamide	GGAFPFRYa	8	912.4607	Am	912.4606	457.2375	2	54.26	-0.1
	QFRYa	4	611.3180	Am	611.3179	306.6664	2	30.56	0.5

Note that "a" in the peptide structure represents C-terminal amidation.

Abbrebiations; a.a. : numbers of amino-acid residues, Am: C-terminal amidation, Fr: a fragment of the predicted peptide, Om: oxydation of methionine residue, PTM: post-translational modification, Py: N-terminal pyro-glutamination, RMSE : root mean square error, RT : retension time of nanoLC, Z : electric charge of parent ion.

Supplemental Table 4 (continued)

Name	Structure	a.a	Precise molelular mass					RT	RMSE
			caliculated	PTM	measured	detected (m/Z)	Z	(min)	(ppm)
FVRIamide-1	ISSFVRIa	7	819.4967	Am	819.4966	410.7553	2	48.93	-0.8
	PSSFVRIa	7	803.4654	Am	803.4653	402.7408	2	42.68	2.1
	ASHFVRIa	7	827.4766	Am	827.4766	414.7451	2	32.26	-1.1
	PGNFVRIa	7	800.4658	Am	800.4657	401.2409	2	38.44	1.9
	SSKFVRIa	7	834.5076	Am	834.5076	418.2612	2	31.64	0.3
	GSSFVRIa	7	763.4341	Am	763.4340	382.7279	2	40.85	9.5
	MSSFVRIa	7	837.4531	Am, Om	853.4480	427.7313	2	40.39	0.0
	VSSFVRIa	7	805.4811	Am	805.4810	403.7499	2	43.80	5.3
FVRIamide-2	LSSFVRIa	7	819.4967	Am	819.4966	410.7553	2	48.93	-0.8
	PNSFVRIa	7	830.4763	Am	830.4763	416.2469	2	42.90	3.6
	GSHFVRIa	7	813.4610	Am	813.4609	407.7377	2	32.02	-0.1
	PGNFVRIa	7	800.4658	Am	800.4657	407.7377	2	32.02	-0.1
	ASRFVRIa	7	846.5189	Am	846.5188	283.1801	3	32.92	-0.3
	MSSFVRIa	7	837.4531	Am	837.4531	419.7367	2	45.89	6.9
	IPSSAFVRIa	9	987.5866	Am	987.5865	494.8004	2	49.18	-0.1
	MGQSSFVRIa	9	1022.5332	Am	1022.5331	512.2776	2	46.12	7.3
FXXFamide	AFDRIATSGFARFa	13	1456.7576	Am	1456.7576	729.3914	2	58.68	7.4
	EFDPIAYGQFGKWa	13	1555.7460	Am	1555.7458	778.8871	2	75.05	8.9
	FDRIASVSSFGKFa	13	1458.7620	Am	1458.7620	730.3950	2	57.69	9.3
	FDRIGQSSFRSFS	13	1532.7372	−	1532.7371	767.3820	2	51.50	8.0
	FDRISGLSSFGSFa	13	1417.6991	Am	1417.6990	709.8624	2	69.17	8.0
	FDRISGLSSFGTFa	13	1431.7147	Am	1431.7146	716.8710	2	68.23	9.0
	FDRISGVSGFGRFa	13	1442.7419	Am	1442.7419	722.3840	2	57.83	7.9
	GFDRIGGMSGFSSFa	14	1462.6664	Am	1462.6663	732.3457	2	67.27	7.2
	GFDRIGGMSSFSSFa	14	1492.6769	Am	1492.6770	747.3526	2	67.09	9.1
	GFDRISGMSSFAKFa	14	1547.7555	Am	1547.7554	774.8909	2	63.33	7.7
	GFDRISSASGFAKFa	14	1487.7521	Am	1487.7554	744.8917	2	56.68	9.0
	GIDSINSYSKFGGFa	14	1489.7202	Am	1489.7202	745.8738	2	63.07	8.6
	GLDSIASYGKFGGFa	14	1416.7038	Am	1416.7036	709.3639	2	66.25	6.8
	GLDSIASYSSKFGGFa	15	1533.7464	Am	1533.7463	767.8865	2	64.59	7.9
	GLDSIASYSSQFGGFa	15	1533.7100	Am	1533.7463	767.8793	2	65.30	-1.4
	pEFDPIAYGQFGKWa	13	1537.7354	Am, Py	1537.7427	769.8767	2	84.24	-2.5
	RFDAISSSGFGRFa	13	1444.7212	Am	1447.7212	723.3700	2	57.84	2.9
	RFDRIAGATGFGAFa	14	1483.7685	Am	1483.7683	742.8978	2	59.69	8.6
	RLDSIAPSSTFGSFa	14	1482.7467	Am	1482.7466	742.3817	2	59.34	1.5
	RLDSIASYGKFGGFa	14	1515.7835	Am	1515.7834	758.9041	2	60.18	6.7
	SHDPIGHYGRFGRFa	14	1643.8070	Am	1643.8069	822.9153	2	42.40	5.6
GDPFLRFamide	YLRFa	4	596.3435	Am	596.3434	299.1787	2	43.54	-0.1
	GDPFLRFa	7	849.4498	Am	849.4497	425.7343	2	59.76	5.0
	EEDPDSLEKYLQNGGYLRFa	19	2271.0808	Am	2271.0808	758.0417	3	78.35	9.9
	NVKDGGFLRFa	10	1150.6248	Am	1150.6248	576.3232	2	52.23	6.3
	SDVDLPFGNKHYLHFa	15	1786.8792	Am	1786.8790	894.4529	2	58.70	6.9
	NYLRFa	5	710.3864	Am	710.3864	356.2004	2	42.57	-0.2
GnRH-RP	TKAQHYHFSNGWHPa	14	1707.8019	Am	1707.8019	570.2742	3	32.80	-0.7

Supplemental Table 4 (continued)

Name	Structure	a.a	Precise molelular mass					RT	RMSE
			caliculated	PTM	measured	detected (m/Z)	Z	(min)	(ppm)
HCS2	AVFYTQSNNNDFPRLa	15	1783.8642	Am	1783.8643	892.9410	2	59.75	1.8
	SVFTASADGVYPRIa	14	1480.7675	Am	1480.7673	741.3924	2	57.47	2.0
	GIFTAGGPGFPRIa	13	1287.7088	Am	1287.7087	644.8678	2	67.10	9.6
Helicostatin-RP	WGSLGIGNW	8	988.4767	−	988.4767	495.2492	2	80.34	7.3
	WGSLGLSSRYRa	11	1279.6786	Am	1279.6785	427.5670	3	46.79	0.4
	WGVLGLGSRIKWGPSLRa	17	1880.0898	Am	1880.0896	627.7047	3	64.24	1.4
	LGRLELEa	7	827.4866	Am	827.4865	414.7531	2	43.88	6.2
Leucokinin	KAFSVWAa	7	806.4440	Am	806.4439	404.2308	2	45.06	4.0
	SANTRTAASARPa	12	1200.6324	Am	1200.6323	601.3233	2	19.62	-0.2
MAP-5A	RTLGLGNKEFADMLH	14	1700.8669	Am	1716.8618	573.2945	3	43.09	-0.1
MAP-6B	EAVAGKDHTETGA	13	1284.5947	−	1284.5945	643.3043	2	16.18	-0.3
MAP-8	AFKNDNFQRLPY	12	1511.7522	−	1511.7520	756.8840	2	46.93	1.0
MIP-1	RHAPLFIa	7	851.5130	Am	851.5129	426.7646	2	38.37	2.0
Myomodulin	GIQMLRLa	7	828.5004	Am	828.5004	415.2607	2	54.77	7.9
	ALDYRRLARGIQMLRLa	16	1943.1364	Am	1943.1362	648.7249	3	68.23	8.6
	pQLPLLRLa	7	833.5453	Am	833.5486	417.7848	2	82.40	7.6
	PMGMLRLa	7	815.4510	Am	815.4510	408.7357	2	52.66	7.2
	PVGMLRLa	7	783.4790	Am	783.4789	392.7496	2	53.39	7.4
Orcokinin-RP	SFDPISRTGVMGSFGa	15	1555.7454	Am	1555.7452	778.8875	2	65.08	9.8
Pedal peptide	SFDSINGNSKLNSFA	14	1599.7529	−	1627.7590	814.8943	2	57.11	9.2
	PFDSISGNSGLSSFA	14	1484.6784	−	1484.6783	743.3461	2	109.88	-0.4
	PFDSINGNSRLSAFA	14	1594.7740	−	1594.7739	798.3995	2	60.94	6.6
	PFDSISGNSRLSAFA	14	1567.7631	−	1567.7629	784.8895	2	60.72	1.0
PentaFVa-1	LRDFVa	5	647.3755	Am	647.3755	324.6954	2	34.39	1.7
	HRYFVa	5	719.3868	Am	719.3867	360.7007	2	27.02	0.1
	APIREFVa	7	829.4811	Am	829.4810	415.7493	2	41.74	3.8
Pleurin	VFFTRGDKGSSYPRIa	15	1727.91078	Am	1727.9106	864.9640	2	52.90	1.6
SCP-1	SSYLAFPRLa	9	1051.5815	Am	1051.5814	526.8009	2	63.43	5.5
	GYLALPRMa	8	918.5110	Am	918.5109	460.2659	2	57.33	6.8
SCP-2	ASYLAFPRLa	9	1035.5866	Am	1035.5865	518.8040	2	64.11	6.6
Sensorin-A1	RSLGQLDHPEEGVNERLTEAa	20	2248.11967	Am	2248.1123	750.3812	3	39.89	4.2
Sensorin-A2	GRYKMGYMFa	9	1150.54164	Am	1182.5315	592.2736	2	23.83	0.9
SGLVamide	PFGELASGLVa	10	987.5390	Am	987.5339	494.7800	2	68.02	6.6
	PFDNLASGLVa	10	1030.5448	Am	1030.5447	516.2841	2	66.18	8.6
SXFY-1	ARDENSGFY	9	1057.4465	Am	1057.4465	529.7352	2	37.09	8.9
	ARNDNDSFY	9	1100.4523	Am	1110.4521	551.2337	2	34.73	0.6
	ARDDTSSFY	9	1060.4462	Am	1060.4460	531.2308	2	37.42	0.9
	SGDDDLRSFY	10	1173.4939	Am	1173.4939	587.7584	2	55.49	7.1
	SADDHSRFY	9	1096.4574	Am	1096.4573	366.4932	3	34.74	0.4
SXFY-2	GRDDVSRFY	9	1113.5204	−	1113.5203	557.7693	2	41.92	3.4
	APEEHSSFY	9	1065.4404	−	1065.4402	533.7271	2	39.28	-0.5
TDP-1	GFRVNAAGRVAHGYa	14	1472.7750	Am	1472.7749	737.4016	2	36.97	9.3
TDP-2	SSNRVAHGFa	9	972.4860	Am, Fr	972.4890	487.2519	2	20.92	0.3
Tachykinin-1	FHPSAFFGSRa	10	1150.5673	Am	1150.5671	384.5296	3	47.78	-0.1
	FFHQGFVGSRa	10	1179.5938	Am	1179.5938	590.8082	2	40.42	6.9
	FDPSGFTGSRa	10	1128.5353	Am	1069.1425	535.2611	2	44.38	8.3
Tachykinin-2	FIQGFVGSRa	9	1008.5506	Am	1008.5505	505.2867	2	45.89	8.3
	FMPGGYHASRa	10	1120.5237	Am	1120.5236	561.2695	2	32.02	0.8

Supplemental Table 4 (continued)

Name	Structure	a.a	Precise molelular mass					RT	RMSE
			caliculated	PTM	measured	detected (m/Z)	Z	(min)	(ppm)
TERP-1	APTFHGLFVa	9	986.5338	Am	986.5338	494.2775	2	62.30	6.8
	IPDGLAERVPSFGHFRVa	17	1895.0167	Am	1895.0167	632.6796	3	57.41	0.2
	VPSFGHRFVa	9	1043.5665	Am	1043.5664	522.7913	2	41.71	1.6
	IPDNLDIRAPTFGHRFVa	17	1966.0538	Am	1966.0537	656.3594	3	57.91	1.4
	APTFGHRFVa	9	1029.5509	Am	1029.5508	515.7828	2	39.49	0.3
	TFGHRFVa	7	861.4610	Am	861.4609	431.7382	2	32.72	1.1
	APSFGHRFVa	9	1015.5352	Am	1015.5352	508.7751	2	39.65	0.5
	pQPSFGHRFVa	9	1055.5267	Am	1055.5300	528.7759	2	49.78	6.3
	LPTFGHHFVa	9	1052.5556	Am	1052.5555	351.8596	3	47.59	1.3
	VPTFGHHFVa	9	1038.5400	Am	1038.5400	347.1890	3	43.35	5.0
	KPSFGHMFVa	9	1047.5325	Am	1047.5323	524.7738	2	47.60	0.7
	IPSFGHRFVa	9	1057.5822	Am	1057.5822	529.7981	2	47.18	-0.5
	VPAFGHRFVa	9	1027.5716	Am	1027.5715	514.7965	2	43.60	6.7
	VPSFGHMFVa	9	1018.5059	Am	1018.5059	510.2637	2	62.76	6.9
	VPQFGHRFVa	9	1084.5931	Am	1084.5930	362.5403	3	43.35	5.5
TERP-2	QFPSRFQHSFIa	11	1391.7099	Am	1391.7099	696.8672	2	52.43	7.3
	pQFPSRFQHSFIa	11	1374.6799	Am, Py	1374.6832	608.3546	2	64.46	8.2
	RSFFTHRFVa	9	1194.6411	Am	1194.6410	399.2228	3	42.68	4.7
	IPDGLDIRGPGFIHRFVa	17	1907.0530	Am	1907.0530	636.6962	3	66.44	7.2
	GPGFIHRFVa	9	1027.5715	Am	1027.5712	514.7924	2	48.69	-1.3
	PAGFMHRFVa	9	1059.5437	Am,Om	1075.5386	530.7793	2	47.41	0.4
	AGFSHRFIa	8	932.4981	Am	932.4980	467.2567	2	39.49	0.9
	VPLGLEYRARNFHHRFVa	17	2019.1497	Am	2109.1482	704.0569	3	45.70	-0.4
	ARNFHHRFVa	9	1181.6319	Am	1181.6318	591.8225	2	25.60	-1.2
	TPDGFDLRAPGFSHRFIa	17	1930.9803	Am	1930.9788	644.6677	3	59.97	1.2
	DGFDLRAPGFSHRFIa	15	1732.8798	Am	1732.8796	578.6338	3	60.25	-0.1
	APGFSHRFIa	9	1029.5509	Am	1029.5508	515.7846	2	41.92	3.8
	FMSGFNLRGPSFVHRFVa	17	1996.0254	Am,Om	2012.0203	666.3542	3	66.45	7.7
	GPSFVHRFVa	9	1043.5665	Am	1043.5664	522.7924	2	42.77	3.7
	TPDGIDQRAPGFTHRFIa	17	1925.9861	Am	1925.9861	643.0074	3	51.50	7.6
	APGFTHRFIa	9	1043.5665	Am	1043.5665	348.8644	3	41.74	4.8
	IPDGLDIRGPSFIHRFVa	17	1937.0636	Am	1937.0654	646.7003	3	67.52	8.0
	GPSFIHRFVa	9	1057.5822	Am	1057.5820	529.8025	2	50.54	7.8
	AGFSHRFVa	8	918.4825	Am	918.4824	460.2485	2	36.05	0.0
	GSGFMHRFVa	9	1035.5073	Am	1035.5073	518.7607	2	45.60	0.3
	AARYSHRYFa	9	1168.5891	Am	1168.5891	585.3016	2	30.56	-0.2
	APYGLEARAPYHTRFIa	16	1859.9795	Am	1859.9795	621.0052	3	50.34	7.7
	APYHTRFIa	8	1002.5400	Am	1002.5399	502.2767	2	36.43	-1.1
	GFVHKFVa	7	831.4756	Am	831.4755	416.7477	2	38.19	6.3
	GPGFSHRFVa	9	1001.5196	Am	1001.5195	501.7670	2	38.15	0.0
	LSDELDKKASFSHRFVa	16	1876.9796	Am	1876.9795	626.6671	3	41.94	0.0
	ASFSHRFVa	8	948.4930	Am	948.4930	475.2525	2	35.44	-2.7
	SPGFSHRFVa	9	1031.5302	Am	1031.5300	516.7751	2	68.70	5.5
	LDDGIDERGFSHRFVa	15	1760.8595	Am	1760.8595	587.9645	3	50.54	7.0
	GFSHRFVa	7	847.4454	Am	847.4453	424.7298	2	32.20	-0.3

Supplemental Table 4 (continued)

Name	Structure	a.a	Precise molelular mass					RT	RMSE
			caliculated	PTM	measured	detected (m/Z)	Z	(min)	(ppm)
VHGY	GFDDLGGFNVHGY	13	1396.6048	−	1396.6047	699.3159	2	64.84	8.9
	GLDDLGGFRVHGL	13	1354.6994	−	1354.6993	678.3619	2	65.32	7.4
Whitnin	PKYMDT	6	753.3368	−	753.3367	377.6760	2	24.49	1.0
WWamide	WPQFRSWa	7	1004.4981	Am	1004.4980	503.2601	2	64.84	7.5
	GWNQRPFATWa	10	1260.6153	Am	1260.6152	631.3196	2	62.97	7.4
	GWDNRNFATWa	10	1264.5738	Am	1264.5737	633.2983	2	61.93	6.6
	GWDDRSFATWa	10	1238.5469	Am	1238.5469	620.2810	2	63.39	0.5
	GWDDRSFAPWa	10	1234.5520	Am	1234.5220	618.2839	2	66.37	1.0
	GWDDRSFTSWa	10	1254.5418	Am	1254.5305	628.2830	2	65.52	7.7
	PTEWSKLGIWa	10	1214.6448	Am	1214.6448	608.3348	2	70.38	8.5
	pEWNQFLTWa	8	1103.5295	Am, Py	1103.5188	552.7668	2	99.64	0.3
	WKTMATWa	7	921.4531	Am	921.4531	461.7365	2	52.97	5.8
	WSDMKVWa	7	949.4481	Am	949.4480	475.7339	2	56.94	5.4
	WSHVSTWa	7	900.4243	Am	900.4243	451.2210	2	38.76	3.5
	WKSMSVWa	7	921.4531	Am	921.4531	461.7371	2	58.27	7.0
	WKNMAVWa	7	932.4691	Am	932.4691	467.2446	2	56.94	6.0

Supplemental Table 5. Fragment peptides of neuropeptide precursors identified by the nanoLC-Orbitrap MS/MS analysis on *Reisia* ganglia.

Precursor	Sequence	a.a	Position	Precise molelular mass					RT	RMSE
				caliculated	PTM	measured	detected (m/Z)	Z	(min)	(ppm)
APGWa-2	KRAPGWGKRSPVDVDNFDSEDMLNDL	26	59-84	2960.4086	Om	2976.4036	993.1426	3	83.84	0.8
	MLNDLDDDDDADSLEVKRAP	20	80-99	2246.0008	-	2246.0007	1124.0090	2	98.38	1.2
	WGKRSML	7	157-163	876.4640	-	876.4640	439.2429	2	46.81	8.2
ELH	LPTASRSGLATHLEAGEDLAQLLSGPSLAKTLT	33	48-81	3317.7830	-	3317.7830	1106.9358	3	77.00	0.8
	TLTKRDLSINRDLQSLADML	20	79-98	2302.2314	-	2302.2314	1152.1333	2	62.23	8.9
	RAFLRELa	7	113-119	902.5450	Am	902.5450	452.2802	2	35.45	0.9
Fulicin	SSQYEFLGKRPLSLPSH	17	114-130	1945.0057	-	1945.0057	973.5120	2	49.19	1.9
	AVLDYLAKRVLLPYEFVa	17	133-149	2007.1557	Am	2007.1556	1004.5852	2	85.17	0.1
	DSVNHQNIPEVRN	13	183-195	1520.7331	-	1520.7332	761.3741	2	28.82	0.4
	GGPIADQLLRDLVN	14	206-219	1479.8045	-	1479.8044	740.9098	2	77.14	0.4
GGKVa	EMPIGISEVIRRANGa	15	41-55	1639.8828	Am	1639.8828	547.6362	3	68.00	2.4
	FDHTNHEQ	8	94-101	1026.4155	-	1026.4155	514.2152	2	34.48	0.8
ProH4B	AVHNNNMFPALKSLLEDRDVHTG	23	101-123	2577.2758	-	2577.2759	516.4630	5	61.41	1.0
SPa	NSDSKVDLDALKDSLASVEDDLSNFQ	26	44-69	2824.3250	-	2824.3250	942.4510	3	87.19	2.2
	RSLLNNL	7	136-142	828.4817	-	828.4818	415.2457	2	15.67	-5.9
	LTEALDRAANQYYYLQSYQSP	21	163-183	2493.1812	-	2493.1812	832.0734	3	43.35	6.9

Abbreviations; a.a. : numbers of amino-acid residues, Am: C-terminal amidation, Om: oxydation of methionin residue, PTM: post-translational modification, RMSE : root mean square error, RT : retension time of nanoLC, Z : electric charge of parent ion.

Supplemental Table 6. Nuclear receptors and epigenome-related enzymes of *Reishia clavigera* identified by the homology search on assembled clusters.

Category	Name	Query	Tretrieved cluster	E-value	Score
Histon methylation	Protein Arg N-methyltransferase 1A (R-MT1A)	Q99873.3 Protein Arg N-methyltransferase-1 [Homo sapiens]	Cluster-112285.241405_TRINITY_DN2355_c0_g1_i10	3.00E-176	449
	Protein Arg N-methyltransferase 1B (R-MT1B)	Q99873.3 Protein Arg N-methyltransferase-1 [Homo sapiens]	Cluster-112285.111115_TRINITY_DN2355_c0_g1_i11	6.00E-175	449
	Protein Arg N-methyltransferase 2 (R-MT2)	NP_001526.2 Protein Argnine N-methyltransferase-2 [Homo sapiens]	Cluster-112285.200198_TRINITY_DN4422_c0_g3_i1	8.00E-126	379
	Protein Arg N-methyltransferase 3 (R-MT3)	O60678.4 Protein Argnine N-methyltransferase-3 [Homo sapiens]	Cluster-112285.75841_TRINITY_DN163908_c0_g1_i16	7.00E-129	397
	Protein Arg N-methyltransferase 4 (R-MT4)	Q86X55.3 Protein Argnine N-methyltransferase-4 [Homo sapiens]	Cluster-112285.191633_TRINITY_DN6243_c7_g1_i1	0.00E+00	531
	Protein Arg N-methyltransferase 5 (R-MT5)	O14744.4 Protein Argnine N-methyltransferase-5 [Homo sapiens]	Cluster-112285.203702_TRINITY_DN17324_c0_g1_i3	0.00E+00	760
	Protein Arg N-methyltransferase 6 (R-MT6)	Q96LA8.1 Protein Argnine N-methyltransferase-6 [Homo sapiens]	Cluster-112285.178681_TRINITY_DN32146_c0_g1_i2	4.00E-95	293
	Protein Arg N-methyltransferase 7 (R-MT7)	Q9NVM4.1 Protein Argnine N-methyltransferase-7 [Homo sapiens]	Cluster-112285.208557_TRINITY_DN20476_c0_g1_i1	1.00E-168	552
	Protein Arg N-methyltransferase 9 (R-MT9)	Q6P2P2.1 Protein Argnine N-methyltransferase-9 [Homo sapiens]	Cluster-110044.1_TRINITY_DN32467_c0_g1_i10	5.00E-64	263
	Histone Lys methyltransferase (ESET)	Q15047.1 Histone-lysine N-methyltransferase SETDB1,ESET [Homo sapiens]	Cluster-112285.234757_TRINITY_DN45214_c0_g1_i4	2.00E-79	290
	Histone Lys methyltransferase (GLP)	Q9H9B1.4 Histone-lysine N-methyltransferase EHMT1,GLP [Homo sapiens]	Cluster-112285.197800_TRINITY_DN8524_c0_g1_i14	0.00E+00	763
	Histone Lys methyltransferase (MLL1)	Q03164.5 Histone-lysine N-methyltransferase 2A, MLL cleavage product N320 [Homo sapiens]	Cluster-112285.217038_TRINITY_DN602_c5_g1_i3	6.00E-131	430
	Histone Lys methyltransferase (MLL2)	Q03164.5 Histone-lysine N-methyltransferase 2A, MLL cleavage product N320 [Homo sapiens]	Cluster-112285.233458_TRINITY_DN74612_c2_g1_i2	2.00E-101	349
	Histone Lys methyltransferase (MLL3)	Q03164.5 Histone-lysine N-methyltransferase 2A, MLL cleavage product N320 [Homo sapiens]	Cluster-112285.233459_TRINITY_DN26613_c1_g1_i1	3.00E-101	347
	Histone Lys methyltransferase (NSD1A)	O88491.1 Histone-lysine N-methyltransferase, H3 lysine-36 specific, NSD1 [Mus musculus]	Cluster-112285.168350_TRINITY_DN22266_c1_g1_i3	0.00E+00	823
	Histone Lys methyltransferase (NSD1B)	O88491.1 Histone-lysine N-methyltransferase, H3 lysine-36 specific, NSD1 [Mus musculus]	Cluster-112285.97433_TRINITY_DN137831_c0_g2_i5	0.00E+00	822
	Histone Lys methyltransferase (SET1)	O15047.3 Histone-lysine N-methyltransferase SETD1A, hSET1A [Homo sapiens]	Cluster-112285.58185_TRINITY_DN2150_c1_g1_i7	6.00E-86	301
	Histone Lys methyltransferase (SMYD3)	6YUH_A Chain A, Histone-lysine N-methyltransferasee SMYD3 [Homo sapiens]	Cluster-112285.15023_TRINITY_DN189759_c0_g2_i2	2.00E-65	221
Histon demethylation	Histone arginine demethylase (JMJD6)	Q6NYC1.1 Bifunctional arginine demethylase and lysyl-hydroxylase JMJD6 [Homo sapiens]	Cluster-112285.103579_TRINITY_DN1284_c1_g2_i2	7.00E-179	512
	Histon Lysine demethylase (KDM1)	NP_055828.2 KDM-1A lysine-specific histone demethylase 1A isoform b, LSD1 [Homo sapiens]	Cluster-112285.238736_TRINITY_DN8082_c1_g1_i2	0.00E+00	984
	Histon Lysine demethylase (KDM2)	SMS16073.1 Polycomb group protein: Lysine-specific demethylase 2 [Drosophila subobscura]	Cluster-73094.1_TRINITY_DN64139_c0_g1_i6	4.00E-129	432
	Histon Lysine demethylase (KDM3)	CAG2232944.1 KDM3 [Mytilus edulis]	Cluster-112285.196807_TRINITY_DN661_c3_g1_i29	2.00E-157	488
	Histon Lysine demethylase (KDM4)	NP_001073180.1 Lysine-specific demethylase 4D [Rattus norvegicus]	Cluster-112285.182637_TRINITY_DN2296_c1_g1_i4	4.00E-148	471
	Histon Lysine demethylase (KDM5)	Q9UGL1.3 Lysine-specific demethylase 5B [Homo sapiens]	Cluster-112285.177410_TRINITY_DN3905_c4_g1_i10	0.00E+00	1211
	Histon Lysine demethylase (KDM6)	4ASK Lysine-specific demethylase 6b [Homo sapiens]	Cluster-112285.65120_TRINITY_DN94693_c0_g1_i5	3.00E-171	533
Histon acetylation	Histon acetyltransferase (GCN5)	BAB59137.1 GCN5 [Gallus gallus]	Cluster-112285.125541_TRINITY_DN10653_c0_g1_i25	0.00E+00	904
	Histon acetyltransferase (HAT1)	CAG2220445.1 HAT1 [Mytilus edulis]	Cluster-54553.0_TRINITY_DN33793_c0_g1_i3	6.00E-176	499
	Histon acetyltransferase (PCAF)	AAF70498.1 PCAF [Mus musculus]	Cluster-112285.125541_TRINITY_DN10653_c0_g1_i25	0.00E+00	892
	Histon acetyltransferase (ATF2)	NP_001271300.1 cyclic AMP-dependent transcription factor ATF-2 isoform 4 [Mus musculus]	Cluster-112285.238171_TRINITY_DN28169_c1_g1_i5	1.00E-27	118
	Histon acetyltransferase (KAT5)	Q92993.2 Histone acetyltransferase KAT5 (Tip60)	Cluster-112285.41421_TRINITY_DN16782_c3_g1_i1	0.00E+00	533
	Histon acetyltransferase (KAT8)	CAG2231114.1 KAT8 : MOF [Mytilus edulis]	Cluster-41407.0_TRINITY_DN14884_c0_g1_i8	0.00E+00	712
	Histon acetyltransferase (KAT6A)	NP_001292807.1 Histone acetyltransferase KAT6A isoform 2 : MOZ [Homo sapiens]	Cluster-112285.44676_TRINITY_DN7606_c0_g1_i4	7.00E-122	408
	Histon acetyltransferase (KAT2A)	XP_012936868.1 Histone acetyltransferase KAT2A-1 isoform X1 : MORF [Aplysia californica]	Cluster-112285.125541_TRINITY_DN10653_c0_g1_i25	0.00E+00	1090
	Histon acetyltransferase (HBO1)	CAG2245072.1 HBO1 [Mytilus edulis]	Cluster-112285.153602_TRINITY_DN19599_c2_g3_i1	0.00E+00	706
	Histon acetyltransferase (CBP1)	AAL54859.1 CREB-binding protein [Aplysia californica]	Cluster-112285.167402_TRINITY_DN1112_c1_g1_i2	0.00E+00	1293
	Histon acetyltransferase (CBP2)	AAL54859.1 CREB-binding protein [Aplysia californica]	Cluster-112285.237331_TRINITY_DN22515_c1_g1_i4	0.00E+00	1230
	Histon acetyltransferase (SRC1)	Q15788.3 Histone acetyltransferase SRC-1:NCOA [Homo sapiens]	Cluster-66268.0_TRINITY_DN4273_c4_g1_i1	3.00E-54	204
	Histon acetyltransferase (ACTR)	Q9Y6Q9.1 Histone acetyltransferase ACTR:NCOA3 [Homo sapiens]	Cluster-66268.0_TRINITY_DN4273_c4_g1_i1	2.00E-51	196
Histon deacetylation	Histon deacetylase 3 (HDAC3)	NP_990078.2 HDAC3 [Gallus gallus]	Cluster-112285.213501_TRINITY_DN7371_c0_g1_i2	9.00E-164	530
	Histon deacetylase 4 (HDAC4)	XP_046776845.1 HDAC4-1 [Gallus gallus]	Cluster-112285.173534_TRINITY_DN997_c2_g1_i11	0.00E+00	588
	Histon deacetylase 6A (HDZC6A)	NP_001123888.1 HDAC6 [Mus musculus]	Cluster-112285.145487_TRINITY_DN80011_c0_g1_i9	0.00E+00	570
	Histon deacetylase 6B (HDAC6B)	NP_001123888.1 HDAC6 [Mus musculus]	Cluster-112285.78095_TRINITY_DN45969_c3_g1_i18	3.00E-166	514
	Histon deacetylase 8 (HDAC8)	AAF73428.1 histone deacetylase 8 [Homo sapiens]	Cluster-92522.1_TRINITY_DN155824_c0_g1_i10	4.00E-117	363
	Histon deacetylase 10 (HDAC10)	JAR82228.1 HDA10 [Fundulus heteroclitus]	Cluster-112285.223296_TRINITY_DN14206_c3_g1_i6	4.00E-114	371
DNA-methylation	DNA methyltransferase-1A (DMT1A)	XP_005095276.1 DNA methyltransferase-1 isoform X1 [Aplysia californica]	Cluster-112285.278639_TRINITY_DN101054_c0_g1_i2	2.00E-110	363
	DNA methyltransferase-1B (DMT1B)	XP_005095276.1 DNA methyltransferase-1 isoform X1 [Aplysia californica]	Cluster-50683.0_TRINITY_DN117851_c0_g1_i3	3.00E-110	356
	DNA methyltransferase-3 (DMT3)	XP_041350770.1 DNA (cytosine-5)-methyltransferase 3B-like [Gigantopelta aegis]	Cluster-5235.0_TRINITY_DN133400_c0_g1_i1	3.00E-55	194
DNA-demethylation	Methylcytosine dioxygenase-1 (TET1)	OWF54936.1 TET1 [Mizuhopecten yessoensis]	Cluster-112754.0_TRINITY_DN54890_c0_g1_i4	6.00E-17	87
	Methylcytosine dioxygenase-3 (TET3A)	CAC5424230.1 DNA tet3-A, [Mytilus coruscus]	Cluster-112285.18005_TRINITY_DN47200_c0_g1_i2	4.00E-143	489
Ubiquitination	E3 ubiquitin-protein ligase (RINFG1)	NP_002922.2 RING1 (E3 ubiquitin-protein ligase) [Homo sapiens]	Cluster-112285.212609_TRINITY_DN29517_c0_g1_i2	3.0.E-87	278
	E3 ubiquitin-protein ligase (RNF213)	Q63HN8.3 E3 ubiquitin-priteu ligase (RNF213) [Homo sapiens]	Cluster-112285.205588_TRINITY_DN1972_c1_g1_i7.p1	0.0.E+00	1605
	E3 ubiquitin-protein ligase (RNF213α)	A0A0R4IBK5.1 E3 ubiquitin-priteu ligase (enfRNF213-alpha) [Danion rerio]	Cluster-112285.205589_TRINITY_DN19452_c0_g2_i1.p1	0.0.E+00	645
	E3 ubiquitin-protein ligase (TRIM56)	E1BD59.1 E3 ubiquitin-priteu ligase (TRIM56) [Bos taurus]	Cluster-112285.149267_TRINITY_DN8323_c0_g1_i15.p1	2.6.E-29	127
Nuclear receptor	Androgen receptor (AR)	XP_021349228.1 AR-like [Mizuhopecten yessoensis]	Cluster-112285.211187_TRINITY_DN172349_c0_g1_i6	2.00E-40	166
	Estrogen receptor-1 (ER1)	NP_001191648.1 ER [Aplysia californica]	Cluster-112285.31815_TRINITY_DN4655_c4_g1_i5	0.00E+00	532
	Estrogen receptor-2 (ER2)	NP_001191648.1 ER [Aplysia californica]	Cluster-112285.31816_TRINITY_DN251_c0_g1_i8	2.00E-173	513
	Retinoid X receptor-1 (RXR1)	AAU12572.1 RXR [Reishia clavigera]	Cluster-112285.206243_TRINITY_DN29438_c0_g1_i14	0.00E+00	804
	Retinoid X receptor-2 (RXR2)	AAU12572.1 RXR [Reishia clavigera]	Cluster-112285.206242_TRINITY_DN9690_c0_g1_i5	0.00E+00	801
	NGF-receptor (NGFR)	AAB59544.1 NGF-receptor [Homo sapiens]	Cluster-112285.243679_TRINITY_DN13802_c1_g1_i11	4.00E-35	140
	Thyroid hormone receptor-1 (THR1)	P10828.2|THB_HUMAN TH receptor-beta	Cluster-44388.1_TRINITY_DN29164_c0_g3_i1	2.00E-66	230
	Thyroid hormone receptor-2 (THR2)	P10828.2|THB_HUMAN TH receptor-beta	Cluster-112285.165798_TRINITY_DN73475_c0_g3_i1	6.00E-66	229
